# Myonectin and metabolic health: a systematic review

**DOI:** 10.3389/fendo.2025.1557142

**Published:** 2025-07-16

**Authors:** Jorge L. Petro, Jaime Gallo-Villegas, Juan C. Calderón

**Affiliations:** ^1^ Physiology and Biochemistry Research Group-PHYSIS, Faculty of Medicine, University of Antioquia, Medellín, Colombia; ^2^ Research Group in Physical Activity, Sports and Health Sciences (GICAFS), Universidad de Córdoba, Montería, Colombia; ^3^ Sports Medicine Postgraduate Program, and Research Group in Medicine Applied to Physical Activity and Sports-GRINMADE, Faculty of Medicine, University of Antioquia, Medellín, Colombia; ^4^ Centro Clínico y de Investigación SICOR, Medellín, Colombia

**Keywords:** glucose metabolism, hormones, lipid metabolism, metabolic syndrome, obesity, skeletal muscle, type 2 diabetes

## Abstract

Myonectin is a myokine with a potential role in metabolic health. This is a bibliometric and bioinformatics-complemented systematic review aimed to comprehensively analyze the structure, regulation and effects of myonectin on metabolic outcomes relevant to the pathophysiology of chronic metabolic diseases. Fifty-three studies involving cellular, animal, and human models were included. Findings indicate that myonectin is induced by aerobic exercise, nutrients, epinephrin, hypoxia and curcumin but is downregulated by obesity and muscle dysfunction. Evidence suggests that myonectin regulates lipid uptake and distribution across tissues, reduces inflammation and apoptosis and modulates mitochondrial function likely through the activation of AKT and AMP-activated protein kinase (AMPK)-mediated signaling pathways. While most results arising from human studies of good quality are in agreement with animal and cellular data, controversy remains and we discuss challenges and perspectives in the field. In conclusion, myonectin has a diverse role in regulating metabolic health, but a key contribution pertains to lipid regulation, which likely leads to a healthy expansion and distribution of adipose tissue.

## 1 Introduction

Skeletal muscle is a tissue with notable plasticity, capable of adapting to a range of physiological stimuli (1–4). This adaptability is not only essential for movement and performance but also plays an important role in metabolic health. In the context of the pathophysiology of chronic metabolic diseases, such as obesity, metabolic syndrome (MS) and type 2 diabetes (T2D), skeletal muscle plays an integral role in storing and oxidizing glucose and lipids, thus significantly impacting the regulation of global energy homeostasis and insulin sensitivity (5–8). Therefore, the functional status of skeletal muscle is a key determinant of metabolic health and should be improved as an essential component in the management of several chronic metabolic conditions (8).

Another feature of skeletal muscle is its ability to function as an endocrine organ through the secretion of myokines, which are a variety of molecules, mainly peptides and proteins, with autocrine, paracrine, and endocrine roles (9). Myokines modulate numerous physiological processes, including metabolism, cardiovascular function, inflammation and oxidative stress (8, 10–14). Over the greater than 200 myokines identified to date (15–17), some have gained attention for their potential roles in driving pathophysiological events of common metabolic conditions such as obesity, MS and T2D (8, 18, 19), and by a proxy, their potential as therapeutic targets. A prominent example is myonectin, which has been recognized for its regulatory effects on lipid metabolism (20, 21).

Myonectin is a 354-residue protein with a mass of 37.28 kDa in humans (UniprotKB Q4G0M1) and 340 residues with a mass of 36.27 kDa in mice (UniprotKB Q6PGN1). It is encoded by the erythroferrone (*ERFE*) gene, primarily expressed in skeletal muscle (20). It has been observed that both exercise and the ingestion of substrates such as lipids increase the expression of myonectin; conversely, obesity induced by a high-fat diet (HFD) decreases its expression in skeletal muscle and circulating levels in animals (20). Increased concentrations of myonectin have been shown to reduce the extracellular levels of free fatty acid (FFA) by favoring their uptake in adipocytes and hepatocytes in cellular and murine models (20, 22). Furthermore, myonectin is reported to activate signaling pathways related to inflammation, metabolism, and mitochondrial biogenesis (22–24).

Human studies evaluating the relationship between myonectin and metabolic variables have yielded contradictory results (21). While some studies report a negative correlation between myonectin and clinical outcomes such as body mass index (BMI), body fat percentage, and insulin resistance (IR), others have found positive correlations (21, 25–27). Additionally, an increase in the levels of myonectin has been reported following aerobic exercise in obese individuals (28), although significant changes have not been observed in all cases (25, 29). Overall, the discrepancies in results between animal and human studies and also among human studies underscore the need to comprehensively survey the role of myonectin across various metabolic contexts and experimental models (*e.g*., cellular, animal, or human).

Here, we rigorously assess, synthesize and integrate current knowledge on the structure, regulation and effects of myonectin on metabolic outcomes relevant to the pathophysiology of obesity, MS, and T2D, through a systematic review of molecular, cellular, animal, and human studies. Bioinformatics and bibliometric analyses provided additional information to address the aim of the review.

By summarizing the available evidence, we identify gaps in knowledge and propose perspectives that delve into the mechanisms and clinical implications of myonectin in the context of the above chronic metabolic disorders.

## 2 Methods

### 2.1 Bioinformatics and bibliometric analyses

The bioinformatics analysis focused on protein family classification, functional annotations and the structural modeling of myonectin. For that, the FASTA sequences for human (UniProtKB Q4G0M1), mouse (UniProtKB Q6PGN1) and rat (UniProtKB D4AB34) myonectin were first retrieved from the UniProt database (30). UniProt, InterPro, and Gene Ontology databases were then used for the corresponding annotations, and Jalview v2.11.4.0 was used for the multiple sequence alignment of the human, mouse, and rat proteins. To predict the three-dimensional structure of human myonectin, the FASTA sequence of the human protein was submitted to the I-TASSER server (v2024) for *de novo* modelling using its default pipeline (template threading, replica-exchange Monte Carlo assembly, and atomic-level refinement) (31). In parallel, the pre-computed AlphaFold2 model (model AF-Q4G0M1-F1-v4) was downloaded from the AlphaFold Protein Structure Database (32, 33). Both procedures were entirely carried out *in silico*, relying on implicit solvent models for relevant energy evaluations, and were performed without the addition of external experimental constrains. For a complementary evaluation of the quality of the predicted structures, the models were analyzed using the Swiss-Model structure assessment tools (34). This analysis focused on the QMEANDisCo global score as an estimate of folding reliability, the MolProbity Score, the clashscore, and the Ramachandran plot statistics. Finally, after water molecules and hetero-atoms were removed, both structures were superposed in UCSF ChimeraX v1.7 using the “matchmaker” tool (Needleman-Wunsch, BLOSUM-62, gap-open 12, gap-extend 1), and the root mean square deviation (RMSD) was calculated over Cα atoms.

On a different bioinformatics analysis, changes in gene expression induced by exercise were evaluated using the Meta-analysis of Skeletal Muscle Response to Exercise (MetaMEx) databank (35). Moreover, putative human small non-coding ribonucleic nucleic acids (miRNA) targeting the 3′ untranslated region (3′UTR) of the human *ERFE* transcript were predicted using TargetScanHuman v8.0 (https://www.targetscan.org/vert_80) and miRDB (https://mirdb.org).

For the bibliometric analysis, a Boolean search strategy with the terms “Myonectin” OR “CTRP15” in PubMed was first performed. Given the relatively small number of publications on myonectin, the bibliometric analysis was applied to the entire set of retrieved articles, with the aim of highlighting general trends and topics in the field (*i.e.*, without being limited by the inclusion criteria of the systematic review). The records were downloaded from PubMed in comma-separated values (CSV) files. As only one database was used, no duplicates were expected, however, all entries were manually checked to ensure completeness and consistency of metadata prior to bibliometric analysis (36). To improve the accuracy of the analysis, a thesaurus was used to merge equivalent terms (*e.g.*, “skeletal muscle” and “muscle, skeletal”) under a single label; the analysis included all available keywords (both author and MeSH terms, when applicable), which broadened the terminological coverage and facilitated the identification of conceptual links and emerging, non-standardized terms. The final database fed VOSviewer v1.6.20 and Bibliometrix for R v4.4.1 softwares to generate a keywords co-occurrence network map and to graph a factorial analysis word map, respectively. The co-occurrence map identifies clusters and networks established in the field by identifying how many times two or more key words appear together in the same set of papers (36). The size of the nodes illustrates the frequency of the occurrence of the keywords and their association with other nodes is represented by the quantity of connections (links). The number of links, the overall strength of these links (total link strength), and the frequency of each term in the analyzed literature (occurrences) is quantified to give a result of the form (links; total link strength; occurrences). Higher values reflect the respective relevance of a term and its integration within the network. The factorial analysis complements the landscape of the conceptual structure of the field. This method extracts a common underlying set of new factors (Dimensions) that explain the variability and relationships among the elements of the network, clearly separating potential clusters and informing about the distance between those clusters. Each cluster is highlighted in one single color. These analyses allow for the mapping of knowledge structures and identification of topics more frequently addressed within the field. They also help to identify emerging subfields, biases or gaps, while simplifying the understanding and visual inspection of a large set of data. Finally, ResearchRabbit (https://www.researchrabbit.ai) was utilized to create a connection map of articles based on the descriptor “Myonectin”, allowing visualization of the citation network and identification of the most relevant authors in the field and the most influential publications.

### 2.2 Systematic review of the literature

### 2.3 Protocol and registration

This review was carried out in accordance with the framework established by the Preferred Reporting Items for Systematic Reviews and Meta-Analyses (PRISMA) guidelines (37). Given its design to integrate information from multiple experimental models (*i.e.*, from basic sciences through to epidemiological studies), no *a priori* registration was conducted.

### 2.4 Information sources

The searches were performed in three major databases: PubMed, ScienceDirect and Scopus. Additionally, a manual search was conducted in Google Scholar and ResearchRabbit to identify any relevant studies not captured by the databases.

### 2.5 Search strategy

The search strategy included the use of the key descriptor “Myonectin” and the Boolean search “Myonectin” OR “CTRP15”. Only original articles, in English, were included. No constraints were imposed on cell line, animal species or strain, human population or setting, geographic area, age, sex or ethnicity. The search range covered from the publication of the first article on myonectin in 2012 up to April 30^th^, 2025.

### 2.6 Eligibility criteria

The systematic review included peer-reviewed original articles that: (i) included any molecular approach or were conducted in cellular or animal models, or humans, with any design (*i.e.*, experimental, observational, clinical trials, *etc.*); (ii) evaluated the effect of regulators on myonectin levels in blood or any tissue; (iii) assessed the effects of myonectin and/or its mechanisms of action; (iv) studied the relationship of myonectin with metabolic outcomes in models of obesity, MS, or T2D in animals or humans, particularly those associated with glucose metabolism, lipid metabolism, body composition, mitochondrial function and inflammatory markers. Articles were excluded if: (i) it was not possible to access the full text article for an evaluation of the methodology and results; (ii) they were not original papers (*e.g.*, reviews, editorials, thesis, and grey literature); (iii) they involved studies focused on other pathologies such as cancer, hematological disorders, sleep apnea syndrome or polycystic ovary syndrome (PCOS); (iv) the studied population was limited to children or adolescents; (v) they were not published in English.

#### 2.6.1 Study selection

After removing duplicates, the study selection followed a two-step process. First, one author (JLP) evaluated the titles and abstracts to exclude studies that did not meet the eligibility criteria. Second, the full-text eligibility of the remaining studies was assessed by two authors (JLP and JCC). Any discrepancies were resolved through discussion among all authors (JLP, JCC, and JGV).

### 2.7 Data collection process

Data extraction was carried out independently by two authors (JLP and JCC) using a standardized format, designed to collect specific information according to the study model: cellular, animal and human. For cellular models, information was extracted about the authors, the cell type, treatment with recombinant myonectin (*i.e.*, concentrations and organism in which it was produced), and the main findings or mechanisms unveiled. In the case of animal models, information was collected about the authors, the animal species, the myonectin measurement technique, the sample or tissue where myonectin was measured, details of the intervention or condition studied (*e.g*., myonectin concentration, diet, exercise), and the main findings. For human studies, information was recorded about the authors, sample size, age, BMI and participant characteristics, study design, the myonectin measurement technique, the intervention conducted, and the main findings. Any discrepancies in data extraction were resolved through discussion and consensus between the two authors (JLP and JCC). When necessary, a third author (JGV) was consulted to resolve any disagreements.

### 2.8 Risk of bias assessment

To assess the risk of bias in the included studies, quality assessment tools tailored to each type of study model were used. The SYRCLE’s Risk of Bias Tool (38) was employed independently by JLP and JCC to assess animal studies. For randomized controlled trials (RCT) in humans, the Cochrane Collaboration’s Risk of Bias 2 (RoB 2) (39) was applied independently by JLP and JAG. The evaluation criteria asked for the following potential biases: selection (was the allocation sequence well generated and concealed? Were the groups comparable at baseline or were the proper adjustments applied in the statistical analysis?), performance (was random housing and blinding to caregivers applied? Were there deviations from the intended interventions?), detection (were outcomes randomly assessed by a blinded researcher?), attrition (were incomplete data adequately addressed?) or reporting biases (are the reports free of selective outcome reporting?). Any other potential bias raised by the authors according to their disciplinary knowledge and expertise was also verified. Initial Kappa indexes over 0.8 were calculated for the concordance for each pair of evaluators. The remaining few discrepancies in the risk of bias assessment were then resolved through discussion between the reviewers and the intervention of a third reviewer was not required in any case.

### 2.9 Bias across studies

Bias across studies was primarily assessed by reviewing the characteristics of the published studies that met the inclusion criteria. An active search for unpublished data was not conducted, as this review focused on published and accessible literature. This approach aligns with the specific objectives of the review and the purpose of basing conclusions on evidence that has undergone peer review.

### 2.10 Synthesis of results

The results of the included studies were synthesized descriptively, as no meta-analysis was conducted. Tables were prepared to summarize the findings related to the metabolic regulators of the expression of myonectin and those related to the effects of myonectin on metabolic outcomes according to the cellular, animal, and human models. Furthermore, the consistency and biological plausibility of these findings were analyzed across models, and possible explanations for the observed variations among studies were explored.

### 2.11 Certainty assessment

**Figure 1:**
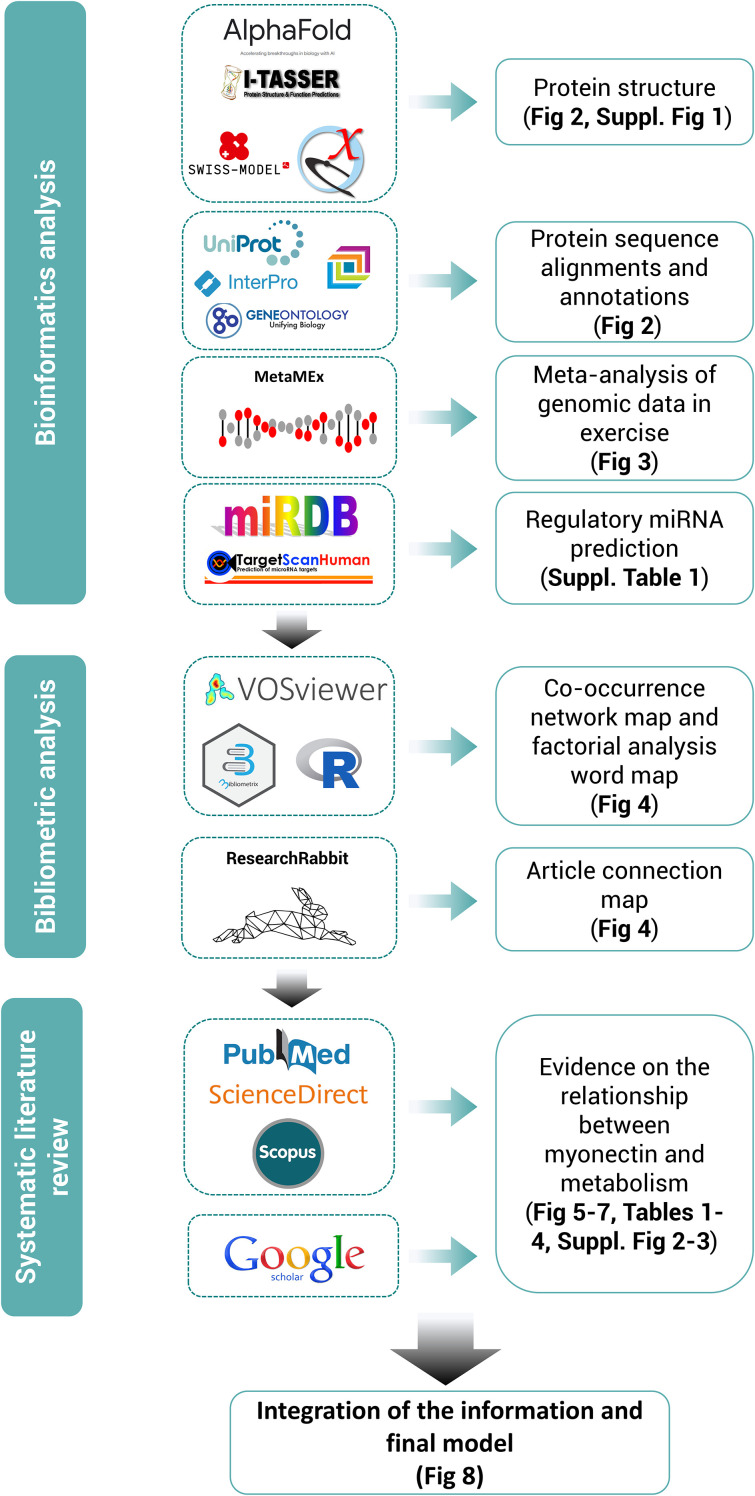
Methodological design of the systematic review following PRISMA guidelines, supported by bioinformatics and bibliometric analyses. Tools used for each section of the review are highlighted along with their outcomes.

The certainty of the evidence was assessed by critically reading the articles and appraising specific methodological aspects, according to the study model: design, sample size, comparability of the groups at baseline, appropriate use of controls, adherence to guidelines for the reporting of the results, validity of the analytical techniques and statistical approaches used, *etc.* Then, the consistency of the results was assessed based on the precision of the estimated effects and the quality of the figures. The direct relevance of the studies to the research questions of this review was also considered. This critical evaluation helped to underpin the strength of the conclusions and study perspectives derived from the findings of the review. **Figure 1** outlines the methodological design of this systematic review.

## 3 Results

### 3.1 Bioinformatics analysis: structural and functional annotations of myonectin

**Figure 2:**
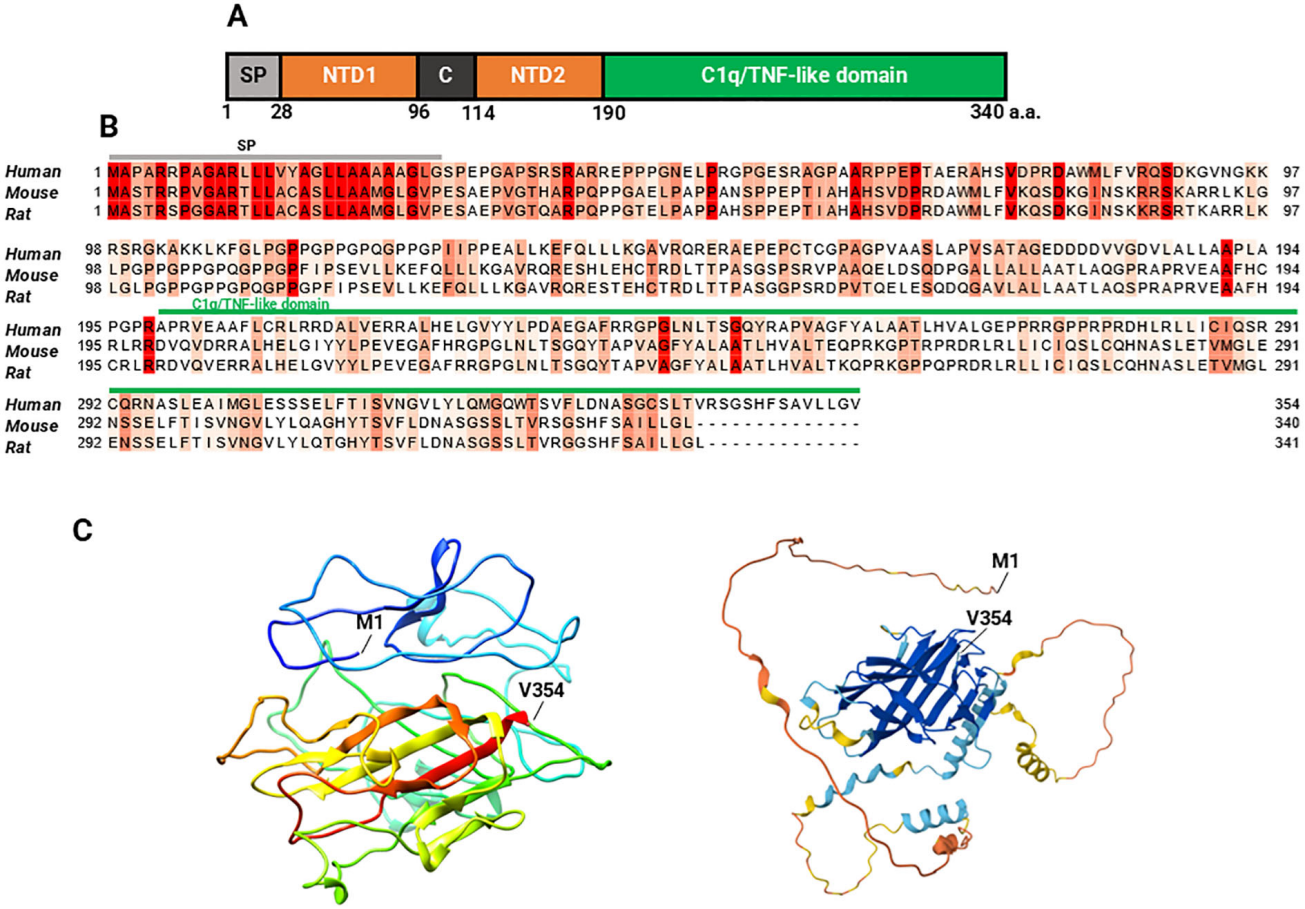
Myonectin sequence and structure. Domain structure of mouse myonectin: **(A)** The five domains of mouse myonectin are shown in different colors: the signal peptide (SP), two amino-terminal domains (NTD1 and NTD2), the collagen-like domain C, and the C1q/TNF-like domain, which is characteristic of the CTRP family which myonectin belongs to. In its structure, it has four potential N-linked glycosylation sites (N229, N281, N292, and N319), and four cysteine residues (C142, C194, C273, and C278) (not marked). Scheme and description of the scheme based on the references (20, 41). **(B)** Multiple sequence alignment of myonectin between humans (Q4G0M1), mice (Q6PGN1), and rats (D4AB34) highlights the conserved regions across the three species (labelled in red), as well as the specific variations. **(C)** On the left, the three-dimensional model of human myonectin generated using I-TASSER is shown (C-score = -2.40, estimated TM-score = 0.43 ± 0.14, and estimated RMSD = 12.3 ± 4.4 Å). On the right, the predicted structure of human myonectin using AlphaFold is shown, which generates a per-residue model confidence score (pLDDT) ranging from 0 to 100, represented by color: 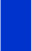 very high (pLDDT > 90), 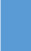 high (90 > pLDDT > 70), 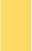 low (70 > pLDDT > 50), 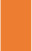 very low (pLDDT < 50). M1 indicates the location of the methionine in position 1 (N terminal) and V354 indicates the valine in position 354 (C terminal). A comparison between the human structures predicted by I-TASSER and AlphaFold and can be seen in the **Supplementary Figure 1**.

Myonectin is a myokine expressed at high levels in skeletal muscle and, at much lower levels, in other tissues such as the lung, eye, smooth muscle, brain, and kidney (20, 40). In skeletal muscles, myonectin expression is highest in muscles with a predominance of oxidative fibers compared to those with predominantly glycolytic fibers (20). The protein is composed of 354 amino acid residues in humans, with a molecular mass (MW) of 37.279 kDa. Its corresponding gene is *ERFE*, also known as *C1QTNF15*, *CTRP15* or *FAM132B*. Myonectin is slightly shorter in mice, with 340 amino acid residues and a MW of 36.265 kDa, and in rats, with 341 amino acid residues and a MW of 36.369 kDa. The protein comprises five domains: a signal peptide for its secretion, the N-terminal domain 1 (NTD1), a short collagen-like domain with six Gly-XY repeats, the N-terminal domain 2 (NTD2), and a C-terminal C1q/TNF-like domain (20). The protein contains four cysteine residues and four potential N-linked glycosylation sites matching the consensus sequence N-X-(Ser/Thr) (**Figure 2**) (20, 41). As expected, the C1q domain is longer in the human protein, spanning positions 199–354. The protein belongs to the tumor necrosis factor TNF-like superfamily and forms homodimers linked by disulfide bonds, but it can also assemble into trimers, hexamers, and oligomers with a higher MW (20). Furthermore, it can form heteromeric complexes with complement C1q tumor necrosis factor-related proteins (CTRP) 2 and 12 (C1QTNF2 and C1QTNF12), and to a lesser extent with CTRP5 and CTRP10 (20).

Sequence alignment reveals 74.12% identity between human and mouse myonectin, 73.31% identity between human and rat myonectin, and 93.22% identity between mouse and rat myonectin (**Figure 2**), indicating partial conservation across these species.

**Figure 2** shows predicted structural models of myonectin, as an experimentally determined structure has not been published. Both models predict a well-defined globular core enriched in β-sheets, surrounded by a few short α-helices. The apparent volume of the protein is increased by three long, flexible, and less ordered loop regions, whose likely intrinsically disordered nature may explain the poor performance of the models in these areas. In line with these structural features, the I-TASSER model obtained a QMEANDisCo score of 0.37 ± 0.05 according to the Swiss-Model evaluation, a MolProbity Score of 3.25, a Clash Score of 8.31, and a concerning 18.97% Ramachandran outliers. The AlphaFold2 model exhibited a global QMEANDisCo score of 0.42 ± 0.05, a MolProbity Score of 1.99, and a Clash Score of 1.13, although with 10.51% of residues located in outlier regions of the Ramachandran plot. These global indicators suggest that, although both models showed modest scores and a considerable number of backbone geometry issues, the AlphaFold2 model performed better overall.

**Figure 3:**
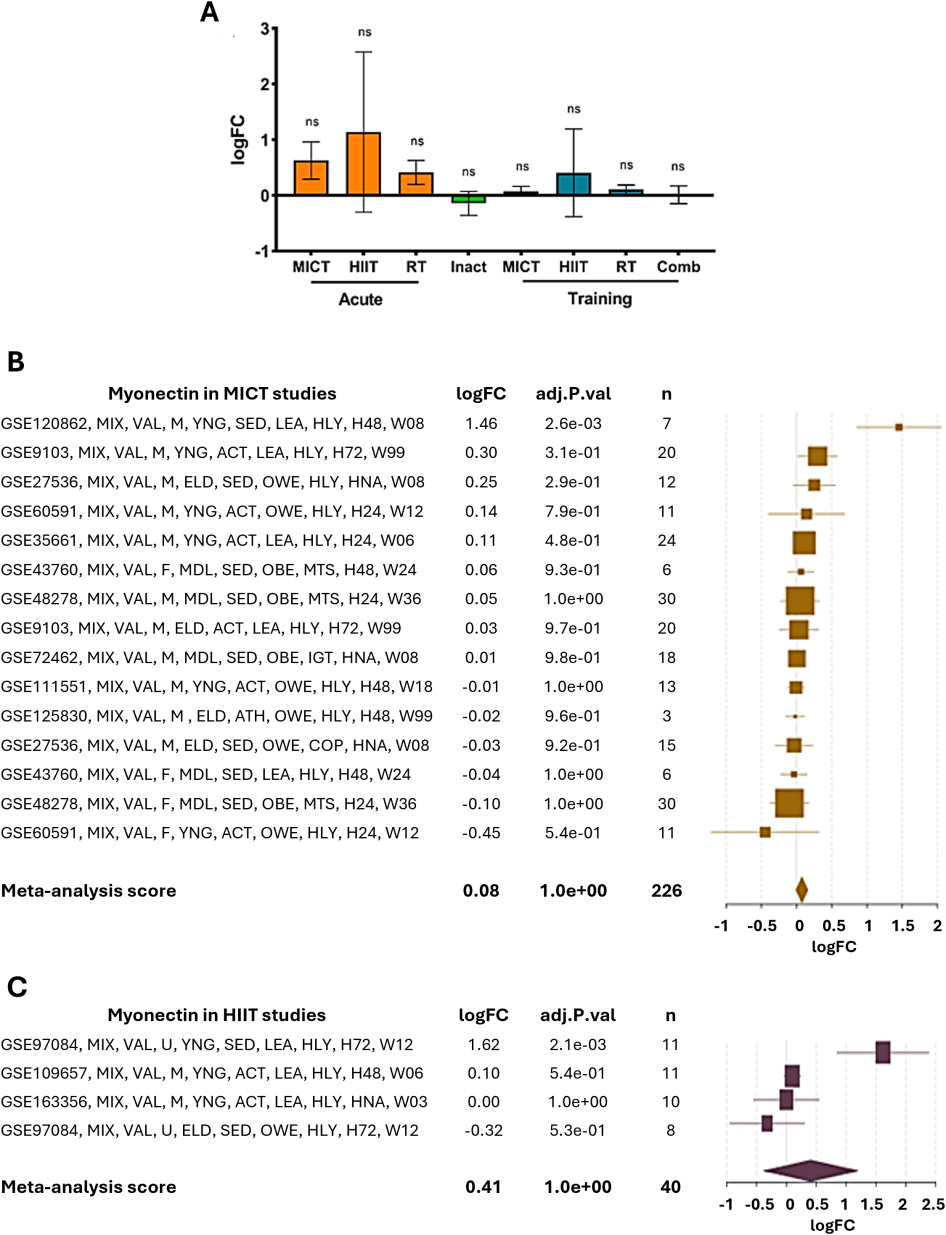
Effect of exercise on myonectin gene expression in humans. **(A)** The effect of different types of physical exercise is presented. Acute: Moderate intensity continuous training (MICT, n=34), high-intensity interval training (HIIT, n=25), and resistance training (RT, n=90); inactivity (Inact, n=115); Training: MITC (n=226), HIIT (n=40), RT (n=213) and combined training (Comb, n=71). ns, not significant. **(B, C)** As above Effect of MICT and HIIT, respectively, on myonectin gene expression. The studies are listed on the left, with logFC (log fold change) values and adjusted p-values (adj.P.val) presented in the adjacent columns. The size of the squares in the graph on the right reflects the sample size (n), while the horizontal bars indicate the confidence intervals. Studies showing positive changes in *ERFE* gene expression under exercise are represented at the top, while those with negative or no changes appear at the bottom. The meta-analysis score at the bottom indicates a small and non-significant overall effect of aerobic exercise on myonectin expression. GSE (Genomic spatial event database) accession number, which identifies the specific dataset; MIX, mixed exercise, including concentric and eccentric components; VAL, vastus lateralis muscle, where the biopsy was taken; M, male sex; F, female sex; U, undefined sex; YNG, young age (under 35 years); MDL, middle-age (35–60 years); ELD, elderly (over 60 years); ACT, active participants (regular exercise of more than 150 minutes/week); SED, sedentary participants; LEA, lean participants (BMI <25); OWE, overweight; OBE, obese participants (BMI ≥30); HLY, healthy participants; IGT, impaired glucose tolerance; MTS, metabolic syndrome; H05, biopsy taken 5 hours after exercise; H24, biopsy taken 24 hours after exercise; H48, biopsy taken 48 hours after exercise; H72, biopsy taken 72 hours after exercise; W03, 3 weeks of training; W06, 6 weeks of training; W08, 8 weeks of training; W12, 12 weeks of training; W18, 18 weeks of training; W24, 24 weeks of training; W36, 36 weeks of training; W99, 99 weeks of training.

The transcriptional regulation of the expression of *ERFE* has mainly been studied in relation to exercise in humans. Both moderate-intensity continuous training (MICT) and high-intensity interval training (HIIT) produce a slight, non-significant increase in muscle myonectin expression compared to inactivity (**Figure 3**). Regarding the posttranscriptional regulation, TargetScanHuman did not predict any conserved regions for human miRNA binding in the 3′UTR of the *ERFE* transcript, suggesting a low probability of regulation mediated by evolutionary conserved mechanisms. In contrast, miRDB identified 61 miRNA which may regulate the *ERFE* mRNA, with three of them scoring over 90: hsa-miR-4251, hsa-miR-450a-2-3p and hsa-miR-3619-5p. The top ten hits are listed in **Supplementary Table 1**.

### 3.2 Bibliometric analysis

**Figure 4:**
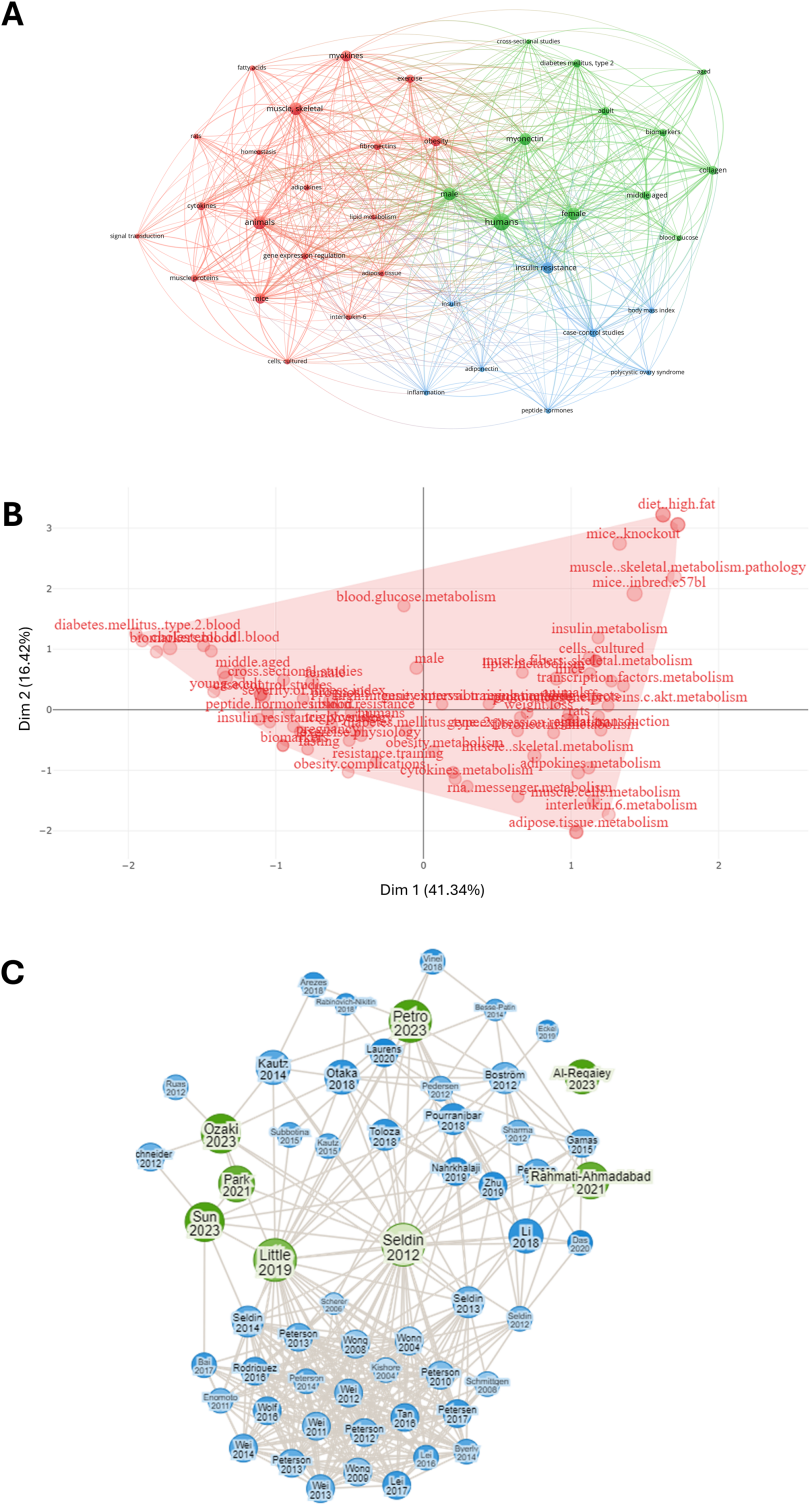
Bibliometric analysis of articles related to myonectin. **(A)** Co-occurrence network map of terms related to myonectin. The nodes represent key terms, while the links show how many times these terms co-appear in the same scientific documents. The colors group the terms into different thematic clusters, highlighting connections between concepts. **(B)** Factorial analysis word map of myonectin-related research terms. The figure shows a factorial analysis of key terms related to myonectin. The axes represent the main dimensions (Dim 1 and Dim 2), capturing 41.34% and 16.42% of the variance, respectively. Keywords such as “diet_high fat”, “metabolism” and “muscle skeletal” are presented as grouped in the dimensional space, showing thematic relationships among myonectin studies. The red area highlights the distribution of these terms on the map, illustrating the interconnection of relevant topics in myonectin research and their associations with metabolism and chronic metabolic diseases. **(C)** Connection map of articles based on the descriptor “myonectin”. The green circles represent the original selected articles, including the classical paper by Seldin et al. (20), while the blue circles indicate articles identified by the software as linked to the selected ones. The size of the circles vary according to the number of connections they have with the green points, providing a visualization of how the studies are interconnected through citations and common themes.

The co-occurrence analysis included 39 items grouped into three clusters (red, green and blue), with 524 links and a total link strength of 1840, indicating a dense and well-connected network characterized by strong semantic cohesion (**Figure 4**). This structure suggests that the literature in this field exhibits a high degree of thematic integration, with clearly defined yet interrelated conceptual areas—potentially reflecting a mature and convergent research domain. The map outlines a continuum that begins with experimental models and culminates in human pathophysiology. The red cluster, the largest in the map with 19 items centers on preclinical research, where studies in *animals* (34; 198; 31), particularly *mice* (34; 136; 19), and in *cell cultures* (23; 46; 6), explore *signaling pathways* (17; 33; 5) and gene regulation in *skeletal muscle* (32; 178; 28). This often happens in the context of *exercise* (27; 78; 13) and its relation to *obesity* (35; 141; 19) or *adipose tissue* (28; 58; 7). The green cluster, comprising 12 items, centers on clinical and epidemiological research involving *humans* (38; 332; 52), with *myonectin* (33; 142; 27) as a key focus. The primary aim appears to be understanding the relationship between myonectin and metabolic alterations, specifically exploring its role in conditions such as *type 2 diabetes* (28; 87; 13) among adult *men* and *women*. Research within this cluster predominantly utilizes *cross-sectional* study designs (24; 45; 5). The positioning of myonectin links it to *obesity* (cluster 1) and *humans* (cluster 2), suggesting a potential role in translating findings on muscle-derived factors, possibly from animal studies in cluster 1, to their relevance in human metabolic diseases. The blue cluster, consisting of eight terms, revolves around *insulin resistance* (33; 167; 25) and related markers (*insulin* [24; 41; 6], *body mass index* [24; 59; 7], and *inflammation* [23; 44; 6]). It also includes *adiponectin* (24; 38; 6) and *peptide hormones* (19; 38; 6), indicating a focus on endocrine networks that modulate insulin sensitivity; the presence of *polycystic ovary syndrome* (15; 41; 6) and *case-control studies* (28; 107; 13) points to the analysis of specific clinical contexts in which *insulin resistance* is pivotal.

Complementarily, the factorial analysis highlighted similar terms and grouped them in one cluster, indicating that beyond the separation of studies according to the experimental models (*i.e.*, preclinical *vs* epidemiological), no further subfields have emerged, and most papers dealt with the same topics and relationships (**Figure 4**).

Finally, the connection map of articles shows that the topic was addressed between 2012 and 2018 by a limited number of groups, led by the research team that discovered the protein. After 2018, the field received contributions from groups all over the world, leading to an expansion in the citation network (**Figure 4**).

### 3.3 Results of the systematic review of the literature: study selection

**Figure 5:**
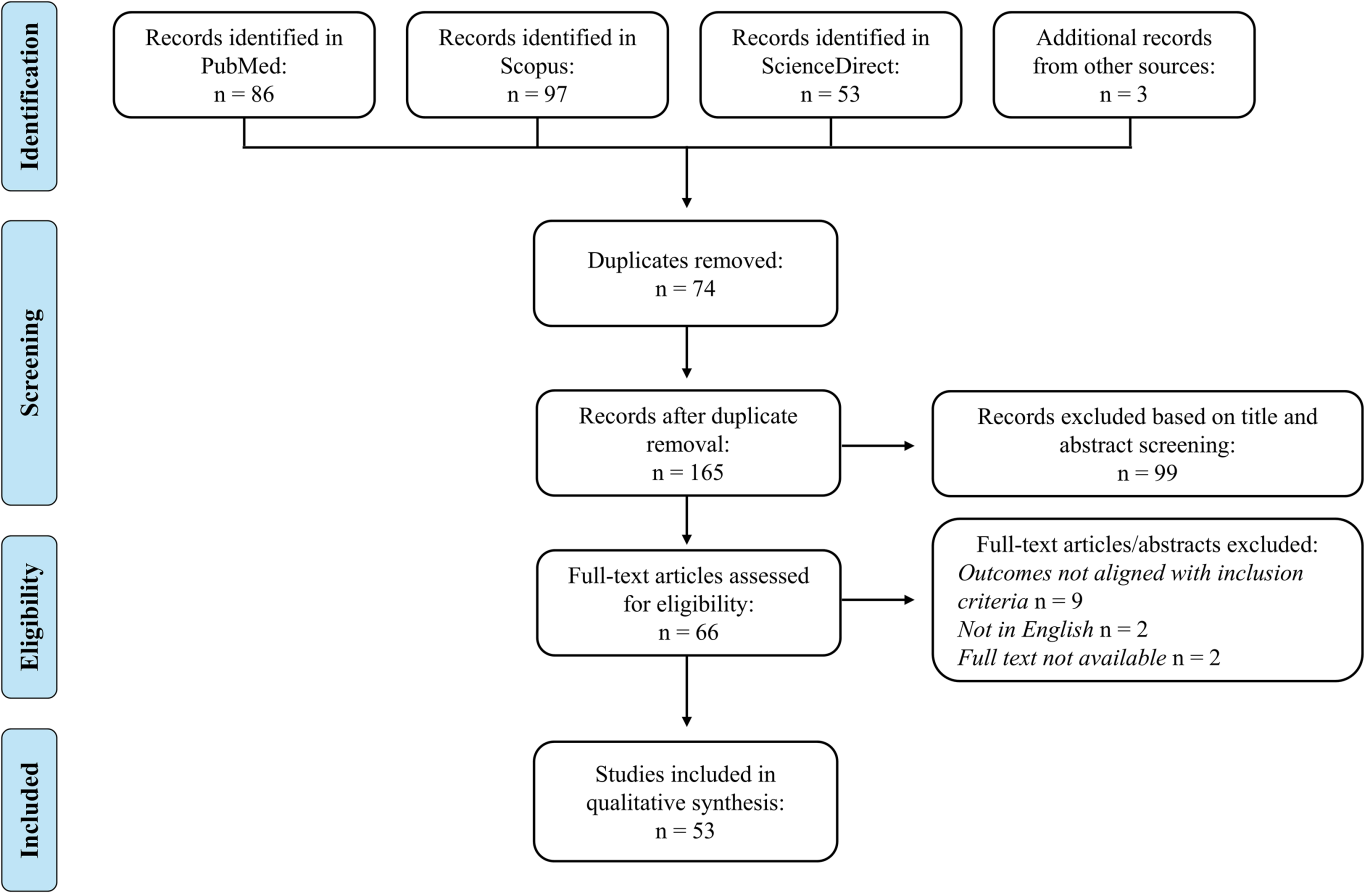
PRISMA flow diagram. A total of 239 records were identified through database searches and other sources. After removing duplicates and screening titles and abstracts, 66 records remained. Full-text articles were assessed for eligibility, leading to the inclusion of 53 studies in the qualitative synthesis.

The study selection process is illustrated in the PRISMA flowchart (**Figure 5**). Initially, a total of 236 records were identified through database searches (PubMed: 86; ScienceDirect: 53; Scopus: 97) and 3 additional records from other sources. After removing duplicates, 165 records were screened by title and abstract, resulting in the exclusion of 99 records; 66 records remained for full-text assessment. Of these, 13 documents were excluded for various reasons, such as not evaluating the outcomes of interest, or the full paper not being available in English. Ultimately, 53 studies were included in the qualitative synthesis.

### 3.4 Studies in cellular models

**Table 1** summarizes the results of 12 studies that explored several *in vitro* mechanisms, such as the uptake of FFA, cell signaling, and mitochondrial markers. This table highlights that the models most used were the C2C12, 3T3-L1 and H4IIE cell lines, exposed to stimuli such as glucose, palmitate or hypoxia, and treated with recombinant myonectin at concentrations typically between 0.5 and 5 µg/mL for up to 24 h.

**Table 1:** Results of myonectin studies in cellular models.

Reference	Model	Recombinant myonectin*	Treatment	Main findings/mechanism
Seldin et al. (20)	C2C12 myoblasts and myotubes; 3T3-L1 adipocytes, H4IIE hepatocytes	⁃ 1, 2.5, 5, or 10 µg/mL for 12 h ⁃ (mouse, HEK 293)	⁃ Glucose (25 mM) for 18 h (C2C12) ⁃ Palmitate (1 μM) for 18 h (C2C12) ⁃ ^3^H palmitate (1 μCi) for uptake of FFA assay (3T3-L1 and H4IIE).	⁃ Myonectin was expressed at higher levels in C2C12 myotubes compared to myoblasts. ⁃ Glucose and palmitate increased myonectin expression in myotubes. ⁃ The up-regulation of myonectin occurred with exposure to epinephrine, ionomycin, and forskolin. ⁃ Myonectin increases the uptake of FFA in adipocytes and hepatocytes by increasing the expression of their membrane transporters (e.g., Cd36, Fabp1, Fabp4). ⁃ Recombinant myonectin has no effect on lipolysis in adipocytes.
Seldin et al. (40)	C2C12 myotubes, H4IIE hepatocytes	⁃ 5 μg/mL for 0.08–24 h ⁃ (mouse, HEK 293)	⁃ Autophagy induced with DMEM 0.1% BSA without glucose, glutamine, or sodium pyruvate (3, 6, 12, or 24 h).	⁃ In C2C12 cells, myonectin expression is reduced with starvation and increased with nutrient availability. ⁃ In H4IIE cells, myonectin decreases the expression of autophagy genes induced by starvation; reduces autophagosome formation and autophagy-mediated p62 degradation; activates the Akt/mTOR pathway.
Yang et al. (44)	C2C12 myotubes		- Palmitate (0.2, 0.4 and 0.6 mM) and oleate (0.6 mM) for 24 h.	⁃ Palmitate-induced insulin resistance is linked to the loss of myotubes and the low expression of myonectin, FNDC5, and FGF21.
Rodríguez et al. (45)	C2C12 myoblasts		- Leptin (10 nmol/L) + irisin (10 ng/mL), applied for 24 h.	⁃ Both leptin and irisin independently increased myonectin expression in C2C12 myoblasts; combined treatment further enhanced expression.
Otaka et al. (23)	Neonatal rat ventricular myocytes, RAW264.7 immune cells, peritoneal macrophages from mice.	⁃ 0.5, 1.0, 2.5, or 5.0 μg/mL for 1 h 0.25–36 h ⁃ (mouse, 293-F)	⁃ Hypoxia-reoxygenation (myocytes) ⁃ Lipopolysaccharide (100 ng/mL) to induce an inflammatory response (macrophages).	⁃ Myonectin reduces cardiomyocyte apoptosis and macrophage inflammatory response through the S1P-dependent activation of the cAMP/Akt pathway, contributing to the improvement of myocardial ischemia-reperfusion injury.
Zhao et al. (46)	Adult mouse cardiac myocytes, NMVCs Cardiac fibroblasts		⁃ NMVCs were transfected with Ad-myonectin or Ad-GFP for 24 h, and adult mouse CFs with Ad-shIR for 72 h to knockdown insulin receptor.	⁃ Myonectin inhibited the differentiation of cardiac fibroblasts into myofibroblasts induced by TGF-β1. ⁃ Reduced expression of profibrotic markers (e.g., collagen I and fibronectin) in fibroblasts treated with myonectin. ⁃ Myonectin inhibited the TGF-β1/Smad3 signaling pathway. ⁃ Myonectin activated the IR/IRS-1/Akt signaling pathway, counteracting the fibrotic effects induced by TGF-β1.
Kawaguchi et al. (47)	RAW264.7 immune cells, primary osteoblasts, and mouse bone marrow cells	⁃ 0.5 µg/mL for 24 h ⁃ (not specified, probably mouse, *E. coli*)	For extracellular flux assays: ⁃ RANKL 75 ng/mL (RAW264.7) ⁃ M-CSF 50 ng/mL and RANKL 75 ng/mL (bone marrow cells)	⁃ Myonectin reduced OCR in RAW264.7 cells. ⁃ Myonectin did not affect OCR levels in osteoblasts. ⁃ Myonectin decreased OCR in bone marrow cells.
Park et al. (48)	3T3-L1 (preadipocyte and adipocyte)	⁃ 0.05-2 µg/mL for 2, or 6 and 10 day (differentiation) or 0.25–48 h (adipogenesis pathways) ⁃ (mouse, *E. coli*)	⁃ Medium of differentiation (0.5 mM methylisobutylxanthine, 1 μM dexamethasone, and 10 µg/mL insulin).	⁃ Myonectin inhibits the adipogenesis of 3T3-L1 preadipocytes by decreasing the expression of adipogenic transcription factors (C/EBPα, β, and PPARγ) and down-regulating the p38 MAPK and CHOP pathways.
Ahmadi et al. (49)	Human THP-1-derived macrophages and primary macrophages from CAD patients and healthy controls	⁃ 1, 5 and 10 μg/mL in primary and THP-1 macrophages, applied for 2 h before LPS (100 ng/mL) stimulation (human, *E. Coli*)	⁃ LPS ⁃ LPS + adiponectin ⁃ LPS + myonectin	⁃ Human myonectin reduced IL-6 and TNF-α secretion in LPS-induced THP-1 macrophages, showing stronger anti-inflammatory effects than adiponectin. ⁃ In primary macrophages, myonectin reduced cytokine expression and secretion only in controls, not in CAD patients.
Takasawa et al., 2022 (50)	Human RD muscle cells, C2C12 Myotubes		⁃ Intermittent hypoxia (64 cycles of 5 min hypoxia and 10 min normoxia for 24 h)	⁃ Intermittent hypoxia significantly increased the mRNA levels of myonectin in both RD and C2C12 muscle cells. ⁃ Myonectin protein levels in culture medium were also increased under intermittent hypoxia. ⁃ The upregulation of myonectin was mediated by OCT1 and NRF2 transcription factors in response to intermittent hypoxia.
Sun et al. (22)	Porcine intramuscular adipocytes	⁃ 5 µg/mL for 24 h ⁃ (Not specified)	⁃ Differentiation with cocktail method. ⁃ Palmitate (30 µg/mL)	⁃ Myonectin inhibits preadipocyte differentiation and increases the expression of FFA transporters FATP1 and FABP4; increases the expression of TFAM, UCP2, and NADH-CoQ, and activates p38MAPK.
Ozaki et al. (24)	C2C12 myotubes	⁃ 5 µg/mL, pretreatment for 1 h ⁃ (mouse, 293-F)	⁃ DEX (100 μM)	⁃ Myonectin ameliorates DEX-induced myotube atrophy through the AMPK/PGC1α pathway.

Ad-GFP, adenovirus carrying green fluorescent protein; Ad-shIR, adenovirus encoding short hairpin RNA for insulin receptor knockdown; Ad-myonectin, adenovirus carrying myonectin gene; Akt, protein kinase B; AMPK, AMP-activated protein kinase; BSA, bovine serum albumin; CAD, coronary artery disease; C/EBPα, β, CCAAT/enhancer-binding proteins alpha and beta; cAMP, cyclic adenosine monophosphate; CD36, cluster of differentiation 36; CFs, cardiac fibroblasts; CHOP, C/EBP homologous protein; C2C12, mouse myoblast cell line; DEX, dexamethasone; DMEM, Dulbecco’s Modified Eagle Medium; *E. coli*, *Escherichia coli*; Fabp1, fatty acid-binding protein 1; Fabp4, fatty acid-binding protein 4; FFA, free fatty acids; FGF21, fibroblast growth factor 21; FNDC5, fibronectin type III domain-containing protein 5; FTP1, fatty acid transport protein 1; HEK 293, human embryonic kidney 293 cells; H4IIE, rat hepatoma cell line; IL-6, interleukin 6; IR/IRS-1, insulin receptor/insulin receptor substrate 1; LPS, lipopolysaccharide; MAPK, mitogen-activated protein kinase; M-CSF, macrophage colony-stimulating factor; mTOR, serine/threonine-protein kinase mTOR; NADH-CoQ, NADH-coenzyme Q reductase; NMVCs, neonatal mouse ventricular cardiomyocytes; NRF2, nuclear factor erythroid 2-related factor 2; OCR, oxygen consumption rate; OCT1, octamer-binding transcription factor 1; PGC1α, peroxisome proliferator-activated receptor gamma coactivator 1-alpha; PPARγ, peroxisome proliferator-activated receptor gamma; p38 MAPK, p38 mitogen-activated protein kinase; p62, sequestosome 1; RANKL, receptor activator of nuclear factor kappa-B ligand; RAW264.7, mouse macrophage cell line; RD muscle cells, human rhabdomyosarcoma muscle cells; S1P, sphingosine-1-phosphate; Smad3, mothers against decapentaplegic homolog 3; TFAM, mitochondrial transcription factor A; TGF-β1, transforming growth factor beta 1; THP-1, Tohoku Hospital Pediatrics-1, human leukemia monocytic cell line; TNF-α, tumor necrosis factor alpha; UCP2, uncoupling protein 2. *Myonectin concentration and incubation time used (source of the sequence, model where the recombinant protein was generated).

### 3.5 Studies in animal models

**Figure 6:**
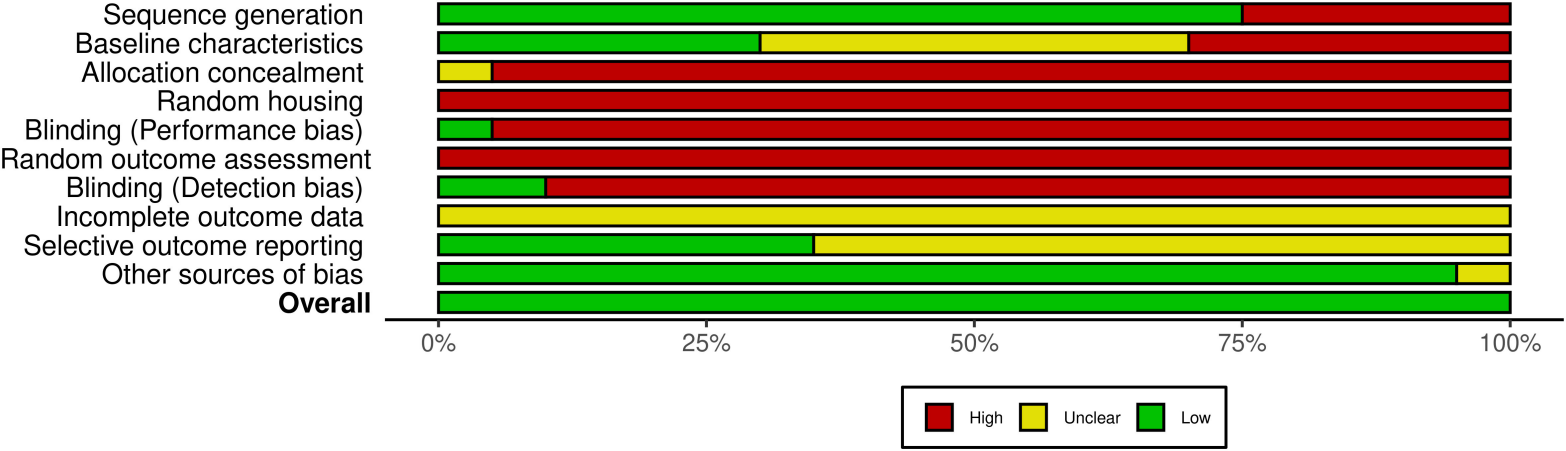
Summary of the risk of bias assessment in animal studies. Summary of the risk of bias across several domains in the 20 studies included, showing the percentage of studies categorized as having high (red), unclear (yellow), or low (green) risk for each type of bias, according to the SYRCLE tool. **Supplementary Figure 2** presents the risk of bias in individual articles.

In this systematic review, 20 animal model studies were included. **Figure 6** shows that the domains with the highest risk of bias for most studies were the allocation concealment, the random housing and outcome assessment, and the blinding for knowing which intervention each animal received and for outcome assessment. By contrast, incomplete outcome data and selective outcome reporting have an unclear risk of bias, largely because the documents did not report on these items. In addition to not adhering to the SYRCLE guidelines, the reviewed animal studies did not explicitly adhere to the more recently issued ARRIVE guidelines (42), which are essential for ensuring transparency and reproducibility in the reporting of results in animal research.

Most of the reviewed studies incorporated HFD and exercise as primary interventions to determine how these conditions modulate the expression and secretion of myonectin. Furthermore, several studies used recombinant myonectin to establish its effect on FFA in plasma in mice, as in the study by Seldin et al. (20). Similarly, genetic models, specifically myonectin knockout mice, were employed to evaluate the physiological consequences of this protein’s deficiency, highlighting the works of Little et al. (43) and Ozaki et al. (24). The most often evaluated metabolic parameters included the expression of genes and the quantity of proteins related to lipid and glucose metabolism in blood, muscle and liver. The results of these studies are presented in **Table 2**.

**Table 2:** Results of myonectin studies in animal models.

Reference	Model (age)	Myonectin measurement technique	Sample/tissue	Intervention/condition	Main findings
Seldin et al. (20)	Male mice C57BL/6 (8 wk)	RT-PCR Western Blot	Soleus and plantaris muscles Serum	⁃ Ex: wheel running (86 ± 29 revs/day) for 3 weeks. ⁃ HFD ⁃ Recombinant myonectin (5 µg/mL).	⁃ Ex increases myonectin expression in muscle and its circulating levels. ⁃ Myonectin reduces circulating FFA levels. ⁃ HFD-induced obese mice have lower muscle expression levels and lower serum concentrations compared to control.
Sharma et al. (51)	Male Fischer-344/Brown Norway rats (9 months)	Western Blot	Plasma	⁃ 6 months of calorie restriction (60-65% of ad libitum intake).	⁃ Calorie restriction reduced fat mass, increased the percentage of lean mass, and improved insulin sensitivity, with no significant changes in myonectin levels.
Seldin et al. (40)	Male mice C57BL/6 (8 wk)	RT-PCR	Muscle (Not specified)	⁃ Fed *ad libitum* ⁃ Fasted for 24 h ⁃ Recombinant myonectin (1 µg/mL).	⁃ Decreased expression (muscle) and serum levels of myonectin due to starvation. ⁃ In liver: myonectin decreases the expression of autophagy genes induced by starvation; activates the Akt/mTOR pathway.
Peterson et al. (52)	Male Zucker rats, obese and lean (6 wk)	RT-PCR Western Blot	Diaphragm muscle	⁃ Ex: treadmill, 5 d/wk, for 9 wk; gradually increased from 10 m/min for 10 min to 20 (obese) - 24 (lean) m/min.	⁃ Myonectin expression tends to be higher in obese rats compared to lean rats. ⁃ Ex decreases myonectin gene expression, while increasing myonectin protein content, regardless of obesity.
Adigozalpour & Safarzade (53)	Wistar rats (10–12 wk)	ELISA	Serum	⁃ Ex: Low volume RT ⁃ Ex: High Volume RT ⁃ Sucrose Control ⁃ Ex: Sucrose Low volume RT ⁃ Ex: Sucrose High volume RT (3 days/week, for 8 weeks).	⁃ Sucrose reduced serum myonectin levels and increased levels of glucose, insulin, and HOMA-IR. ⁃ RT decreased serum levels of myonectin, insulin, and HOMA-IR. ⁃ No correlation was found between serum myonectin and IR in groups fed a normal diet. ⁃ A positive correlation was found in groups fed with a sucrose solution.
Suidasari et al. (54)	Male Sprague-Dawley rats (3 wk)	RT-PCR	Gastrocnemius muscle	⁃ Diet with 1, 7, or 35 mg/kg pyridoxine HCl for 6 weeks	⁃ Myonectin gene expression was significantly higher in the groups that received 7 and 35 mg/kg of pyridoxin HCl compared to the group that received 1 mg/kg; it also showed significant correlations with the expression of Nrf2, HSP60, and myogenin.
Otaka et al. (23)	C57BL/6 heterozygous KO, KO, and myonectin transgenic mice (8–10 wk)	Western blot	Soleus muscle Myocardial tissues	⁃ Ex: Running on a treadmill, 5 d/wk for 4 wk, from 30 to 60 min in duration.	⁃ Increased circulating and muscle myonectin levels from endurance treadmill Ex. ⁃ Ex’s effects on cardiac damage were diminished under myonectin-deficiency condition. ⁃ Myonectin-transgenic mice experienced less severe injury during the ischemia-reperfusion process, with a decrease in apoptotic myocytes in the ischemic myocardium and reduced expression of TNF-a and IL-6 in the ischemic heart.
Little et al. (43)	Female and male myonectin KO and WT mice (5–6 wk)	RT-PCR Western blot	Gastrocnemius muscle	⁃ Ex: Treadmill endurance and sprint test to exhaustion ⁃ Diet: HFD.	⁃ Myonectin deficiency negatively affects blood lipids after an oral lipid load, increases fat storage in adipose tissue, and decreases it in the liver. ⁃ Myonectin is not required for the physiological response to exercise
Jia et al. (55)	Male and female C57BL/6J mice (8 wk)	RT-PCR	Soleus and tibialis anterior muscle	⁃ 24-h fasting *vs* ad libitum feeding.	⁃ Fasting increased myonectin expression in the female soleus and decreased it in the tibialis anterior of both sexes; in fed mice, expression was higher in soleus than in tibialis anterior only in males.
Koohestani Sini et al. (56)	Male Wistar rats (6–8 wk)	ELISA	Serum	⁃ Normal and HFD (12 wk) ⁃ Ex: running on a treadmill at 50 - 60% VO_2max_, five sessions per week for 8 wk.	⁃ Increased serum myonectin levels in the group fed with HFD, which decreased significantly following training.
Gauze-Gnagne et al. (57)	Male Wistar rats (6 wk)	RT-PCR and Western blot	Gastrocnemius	⁃ HFD diets rich in either crude palm oil, refined palm oil, olive oil or lard (12 wk).	⁃ Myonectin gene expression depended on the type of fat used, showing a greater increase with crude palm oil, compared to the refined palm oil and olive oil.
Zhao et al. (46)	Adult and neonatal C57BL/6 ventricles (1–3 days)	Immunofluorescence and immunohistochemical staining, Western blot, and RT-PCR	Heart	⁃ Transverse aortic constriction surgery. ⁃ Tail vein injection of AAV9-CTRP15 or AAV9-EV at day 3 post-surgery.	⁃ Myocardial myonectin expression was reduced in mice subjected to pressure overload. ⁃ Myonectin overexpression reduced cardiac hypertrophy and fibrosis, improving diastolic function.
Rahmati-Ahmadabad et al. (58)	Male Wistar rats with Diabetes (8 wk)	RT-PCR	Soleus muscle	⁃ Ex: HIIT and MICT on treadmill for 6 weeks (5 sessions/wk).	⁃ HIIT and MICT increased myonectin and GLUT4 gene expression compared to the control group, but MICT induced a greater increase compared to the HIIT program.
Koohestani Sini et al. (59)	Wistar rats (6–8 wk)	ELISA	Serum, liver, and muscle tissue	⁃ 12 wk of HFD to induce NAFLD, with 8 wk of HIIT or MICT intervention.	⁃ HIIT and MICT both significantly reduced serum myonectin. ⁃ Myonectin levels in muscle tissue decreased only with HIIT. ⁃ Liver tissue myonectin increased with NAFLD induction but did not significantly change with exercise. ⁃ No significant difference between HIIT and MICT in serum or tissue myonectin levels.
Tan et al. (60)	Male apoE^−/−^ mice, C57BL/6 (8 wk)	Western blot	Aorta Macrophages	⁃ Overexpression of FAM132b ⁃ Western-type diet (12 wk).	⁃ Overexpression of myonectin improved reverse cholesterol transport efficiency; increased circulating HDL-C levels without altering LDL-C, TG, and TC; enhanced cholesterol efflux from macrophages by increasing ABCA1 expression; reduced miR-101-3p expression and increased T-cadherin expression.
Qi et al. (61)	C57BL/6J (4 wk)	RT-PCR and Western blot	Liver, gastrocnemius muscle, WAT, BAT.	⁃ Overexpression of FAM132b (myonectin) (mutations, A136T and P159A) ⁃ HFD (6 wk).	⁃ The treatment with AAV9-Fam132b (A136T and P159A) improved glucose tolerance and insulin sensitivity, in addition to producing a reduction in the adrenergic response to blood glucose elevation and an increase in the adrenergic response to lipolysis in WAT. These changes were accompanied by reductions in body mass, fat mass, and adipocyte size.
Hu et al. (62)	Male Kunming mice (6–8 wk)	ELISA	Quadriceps muscle	⁃ Curcumin supplementation (58 mg·kg^-1^; 28 days; intragastric administration) + treadmill exercise.	⁃ Curcumin increased myonectin content in quadriceps muscle. ⁃ Curcumin reduced fatigue by lowering lactic acid, AMP/ATP ratio, and increasing muscle glycogen, also upregulated PI3K/Akt/AMPK/mTOR pathway, promoting energy metabolism and muscle recovery.
Ozaki et al. (24)	Male C57BL/6 J wild-type, myonectin KO (8–10 or 80 wk old), C57BL/6 mdx, SAMP8, SAMR1/TaSlc (8–10 wk)	RT-PCR	Soleus and gastrocnemius muscles	⁃ Atrophy induced by sciatic nerve denervation and DEX administration.	⁃ Myonectin deficiency accelerates muscle atrophy and weakness in aged male mice and exacerbates muscle atrophy induced by sciatic nerve denervation or steroid administration. ⁃ Myonectin deficiency downregulates AMPK/PGC1α signals and exacerbates mitochondrial dysfunction in denervation-induced atrophic skeletal muscle.
Özçatal et al. (63)	Male Wistar albino rats, control and diabetic (10 wk)	ELISA	Soleus and extensor digitorum longus muscles; serum	⁃ HIIT (4 min 85 - 95% MEC, 2 min 40–50% MEC, 6 cycles, 5 days/wk, for 8 wk).	⁃ Myonectin was significantly higher in the soleus muscle only in the diabetes + HIIT group.
Avcu et al. (64)	Male albino rats (6 wk)	ELISA	Skeletal muscle, liver, kidney	4 weeks of: ⁃ Treadmill exercise ⁃ Energy drink (3.5–10 mL·kg^-1^·día^-1^) ⁃ Exercise + energy drink.	⁃ Increased myonectin in skeletal muscle in all experimental groups vs. control. ⁃ Energy drink group had higher myonectin in skeletal muscle than exercise-only group. ⁃ No significant difference in myonectin levels in the liver and kidney among experimental groups, but the control group had the highest liver myonectin levels. ⁃ Energy drink during exercise increased myonectin in skeletal muscle and kidney, decreased in liver.

AAV9, adeno-associated virus serotype 9; ABCA1, ATP-binding cassette transporter A1; Akt, serine/threonine-protein kinase Akt; AMP, adenosine monophosphate; AMPK, AMP-activated protein kinase; apoE^-^/^-^, apolipoprotein E knockout mice; ATP, adenosine triphosphate; BAT, brown adipose tissue; CTRP15, C1q/TNF-related protein 15; DEX, dexamethasone; d/wk, days per week; ELISA, enzyme-linked immunosorbent assay; EV, empty vector; Ex, exercise; FAM132b, family with sequence similarity 132 member B; FFA, free fatty acids; GLUT4, solute carrier family 2, facilitated glucose transporter member 4; h, hours; HDL-C, high-density lipoprotein cholesterol; HFD, high-fat diet; HIIT, high-intensity interval training; HOMA-IR, homeostatic model assessment of insulin resistance; IL-6, interleukin 6; KO, knockout; LDL-C, low-density lipoprotein cholesterol; mdx, mouse model for Duchenne muscular dystrophy; MEC, maximal exercise capacity; miR-101-3p, microRNA-101-3p; mTOR, serine/threonine-protein kinase mTOR; MICT, moderate-intensity continuous training; NAFLD, non-alcoholic fatty liver disease; PGC1α, peroxisome proliferator-activated receptor gamma coactivator 1-alpha; PI3K, phosphoinositide 3-kinase; RCT, reverse cholesterol transport; revs/day, revolutions per day; RT, resistance training; RT-PCR, reverse transcription polymerase chain reaction; SAMP8, senescence-accelerated mouse prone 8; SAMR1/TaSlc, senescence-accelerated mouse resistant 1; TC, total cholesterol; TG, triglycerides; TNF-α, tumor necrosis factor alpha; VO_2max_, maximal oxygen uptake; WAT, white adipose tissue; wk, weeks; WT, wild-type.

### 3.6 Studies in humans

**Figure 7:**
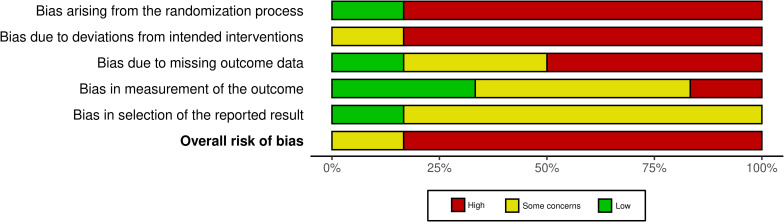
Summary of the risk of bias assessment in human studies. Summary of the risk of bias across several domains in the 6 human randomized controlled trials included, showing the percentage of studies categorized as having high (red), some (yellow) or low (green) risk for each type of bias, according to the RoB 2 tool. **Supplementary Figure 3** presents the risk of bias in individual articles.

A total of 26 human studies were reviewed, of which 20 were observational or non-randomized, and only 6 were RCT, a disparity already recognized in the co-occurrence analysis. The evaluation of the risk of bias of the latter showed a high risk in most studies, particularly in three key domains: the randomization process, the lack of pre-registration of the protocol, and the lack of appropriate blinding of the researchers (**Figure 7**). These aspects contribute to the predominance of a high overall risk of bias across the evaluated studies.

Some observational studies showed that individuals with cardiometabolic disorders (*e.g.*, obesity, MS, and T2D) have higher circulating levels of myonectin compared to controls, and that myonectin is positively correlated with indicators such as BMI, IR, fat mass, and total cholesterol. However, other studies reported opposite results, indicating that people with these conditions have lower myonectin concentrations compared to healthy individuals, as well as negative correlations between myonectin and clinical outcomes related to cardiometabolic disorders (**Table 3**). RCT have focused on evaluating the effect of different exercise modalities on serum myonectin. Findings have been consistent with those of murine models, showing, for example, that mainly HIIT for 8–12 weeks increases myonectin levels in obese individuals. However, no significant changes in circulating levels of myonectin are observed in young, healthy individuals. An increase in myonectin gene expression was found in response to strength training sessions in physically active individuals (**Table 4**).

**Table 3:** Results of myonectin studies in human models with observational and non-randomized controlled trials intervention studies.

Reference	*n*	Age (y)	BMI (kg/m^2^)	Population characteristics	Design	Myonectin measurement technique	Intervention	Main findings
Li et al. (25)	341	nT2D: 56 ± 10 IGT: 55 ± 11 Healthy: 54 ± 9	nT2D: 24.5 ± 3.2 IGT: 24.4 ± 3 Healthy: 23.5 ± 3.1	nT2D, IGT, healthy F and M	Cross-sectional, pre-experimental	ELISA (in plasma)	45 min of treadmill exercise at 60% of VO2_max_, and lipid infusion	⁃ Subjects with T2D and IGT had higher circulating myonectin concentrations than normal subjects. ⁃ A 45-minute exercise period did not change circulating myonectin levels. ⁃ Plasma myonectin does not change significantly with an oral glucose tolerance test, EHC, or lipid infusion (in young individuals).
Toloza et al. (65)	81	30 - 69	F: 27.6 M: 25.8	Subjects with different levels of adiposity and IR, F and M	Cross-sectional	ELISA (in plasma)		⁃ Myonectin levels were positively associated with quartiles of increased area under the insulin curve and negatively with the insulin sensitivity index.
Mi et al. (26)	341	23 – 82	15.6 – 37.6	MS, Healthy, F and M	Cross-sectional	ELISA (in Serum)		⁃ Myonectin was positively correlated with the number of MS components. ⁃ Myonectin concentrations were higher in IR, MS, and obesity.
Kamiński et al. (66)	29	18 – 26	F: 18.5 – 21.3 M: 23.8 – 25.7	Healthy Fand M	Cross-sectional, single-centre study	ELISA (in Serum)	Treadmill test (Bruce protocol)	⁃ Myonectin serum concentration does not change after a bout of exercise. ⁃ Correlation shifted from positive to negative with weight, BMI, and IR markers after exercise.
Li et al. (67)	100	Obese: 36.93 ± 8.31 Controls: 38.05 ± 7.05	Obese: 40.58 ± 4.1, Controls: 24.12 ± 3.14	Obese and healthy, F and M	Longitudinal, pre- and post-LSG (6 months)	ELISA (in serum)	LSG	- Serum myonectin was lower in obese patients compared to controls and increased 6 months post-LSG. - Myonectin showed a negative correlation with BMI, waist circumference, FBG, HOMA-IR, and HbA1c. ⁃ BMI remained the only parameter inversely correlated with myonectin after adjusting for other factors.
Meinecke et al. (68)	11	41.7 ± 6.3	24.8 ± 2.1	Trained M athletes	Uncontrolled intervention study	ELISA (in plasma)	Marathon running	- Myonectin levels significantly increased after the marathon. ⁃ Myonectin levels negatively correlated with fractional shortening post-marathon and 24 h after the race.
Zhang et al. (69)	254	T2D: 57.5–60.2 Controls: 58.6 ± 7.9	T2D: 25.8–26.2 Controls: 25.7 ± 2.9	T2D with normo-, micro-, and macroalbuminuria, and healthy controls; F and M	Cross-sectional	ELISA (in serum)		- Serum myonectin was lower in T2D *vs* controls and decreased progressively from normo- to macroalbuminuria. - It was negatively correlated with BMI, albumin-to-creatinine ratio, LDL-C, TC, BUN, creatinine, uric acid, and positively with glomerular filtration rate and insulin treatment.
Li et al. (27)	362	37 – 64	25.7 ± 3.0	Non-diabetics, T2D, F and M	Cross-sectional	ELISA (in Serum)		⁃ Circulating myonectin levels were lower in individuals with T2D and obesity. ⁃ Myonectin concentrations negatively correlated with metabolic markers of T2D.
Mohassel Azadi et al. et al. (70)	160	T2D: 55 (52–64.5) Controls: 58 (53–66)	T2D: 26.8 ± 4.1 Controls: 26.6 ± 3.9	Participants with T2D and healthy controls; matched by age and sex, F and M	Case–control	ELISA (in Serum)		⁃ Circulating myonectin was significantly increased in T2D relative to controls. ⁃ It showed independent association with BMI and adiponectin in controls, and with HOMA-IR and TNF-α in T2D.
Su et al. (71)	228	Control: 57.22 ± 7.14 T2D without DR: 57.89 ± 9.93 NPDR: 58.54 ± 10.87 PDR: 56.11 ± 8.22	Control: 25.23 ± 1.97; T2D without DR: 25.39 ± 3.74; NPDR: 26.21 ± 4.16; PDR: 25.92 ± 4.27	Control, T2D with/without diabetic retinopathy, F and M	Cross-sectional	ELISA (in serum and aqueous humor)		- Patients with PDR had the lowest serum and aqueous humor myonectin levels, followed by those with NPDR. - Higher myonectin levels were associated with a reduced risk of T2D and DR. - Serum myonectin levels negatively correlated with disease duration, BMI, and HbA1c. ⁃ Aqueous humor myonectin levels negatively correlated with disease duration, systolic, and diastolic blood pressure.
Shokoohi et al. (72)	260	No CAD: 58.45 ± 8.16 CAD: 56.50 ± 8.02	No CAD: 25.67 ± 3.45CAD: 26.59 ± 3.87	With and without CAD, F and M	Cross-sectional (case-control)	ELISA (in Serum)		⁃ Positive correlation between myonectin and BMI, insulin, and FBG, and a negative correlation with HDL-C and adiponectin in patients with CAD
Liu et al. (73)	289	ACS: ≥65 years 9.5% Control: 30.4%	ACS: 25.09 (22.85–27.94) Control: 24.89 (22.78–27.53)	Participants with ACS and healthy controls, F and M	Cross-sectional	ELISA (in Serum)		⁃ CTRP15 lower in ACS compared to controls; independently protective and correlated with coronary stenosis severity.
Petro et al. (21)	90	40 – 60	MS: 30.6 ± 4.0 NMS: 27.0 ± 3.5	Overweight/obesity with metabolic risk (≥1 MS component), F and M	Cross-sectional	ELISA (in Serum)		⁃ Serum myonectin is lower in subjects with MS. ⁃ Myonectin was negatively correlated with the android/gynoid fat mass ratio after multiple adjustments. ⁃ No correlations were found between myonectin and FFA or myosteatosis.
Al-Regaiey et al. (74)	73	T2D: 55.58 ± 0.98 Non-diabetic: 49.97 ± 1.31	T2D: 34.07 ± 0.85; Non-diabetic: 32.13 ± 1.01	T2D and Non-diabetic F	Cross-sectional study	ELISA (in plasma)		- Plasma myonectin levels were significantly lower in T2D. - No difference in myonectin levels between well-controlled diabetics and non-diabetics. ⁃ Myonectin levels were negatively correlated with fasting blood sugar, HbA1c, triglycerides, insulin, and HOMA-IR.
Butler et al. (75)	165	Surgery: 59.0 ± 10.5, Non-surgery: 62.7 ± 9.4, Normal-weight: 48.5 ± 6.4	Surgery: 34.3 ± 7.9, Non-surgery: 43.6 ± 7.5, Normal-weight: 23.6 ± 1.8	Adults with/without bariatric surgery; normal-weight, F and M	Cross-sectional study, 12-year follow-up	ELISA (in plasma)	Bariatric surgery (Roux-en-Y gastric bypass)	- Myonectin levels were lower in both operated and non-operated groups than in the normal-weight group. - No difference in myonectin levels between surgery and non-surgery group; however, despite metabolic improvements, the operated group remained class I obese postoperatively.
Ji et al. (76)	142	Sarcopenia: 72.0 ± 5.7 without sarcopenia: 68.8 ± 6.1	Sarcopenia: 23.2 ± 2.8; without sarcopenia: 26.5 ± 3.1	Older adults with and without sarcopenia, F and M	Cross-sectional	ELISA (in Serum)		⁃ Individuals with and without sarcopenia, with low levels of muscle mass, muscle strength, and physical performance, did not present differences in serum myonectin levels. ⁃ There was no correlation between myonectin and indicators of strength or functional autonomy.
Xue et al. (77)	335	NGT: 53.9 ± 6.2 IGT: 54.9 ± 10.6 T2D 53.6 ± 8.9	NGT: 23.4 ± 2.3; IGT: 24.9 ± 2.4; T2D: 25.2 ± 2.9	Newly diagnosed T2D, IGT, NGT (controls), F and M	Cross-sectional study with exercise test	ELISA (in serum)	OGTT, EHC, lipid infusion, exercise test	- Higher Myonectin and CTRP7 levels in individuals with IGT and T2D compared to NGT. - Serum Myonectin+CTRP7 positively correlates with IR indicators like HOMA-IR, HbA1c, TG, and FFA; negatively correlates with adiponectin. - Myonectin+CTRP7 is a better risk predictor for T2D and IR than Myonectin or CTRP7 alone. ⁃ CTRP7 increases with high insulin and exercise; myonectin shows minimal changes.
Ismail et al. (78)	120	35 – 70		T2D subjects with and without nephropathy, and healthy controls, F and M	Cross-sectional	ELISA (in serum)		⁃ Higher myonectin levels in T2D without nephropathy compared to healthy controls, and lower levels in T2D with nephropathy. ⁃ Significant increase in TG and TC in both T2D groups, and decreased HDL-C levels compared to controls.
Tuama et al. (79)	90	20 - 79	T2D: ≥25 (65%); Con: ≥25 (47%)	T2D and Con (without T2D)	Cross-sectional	ELISA (in serum)		⁃ Circulating myonectin levels were lower in T2D patients. ⁃ Myonectin levels were negatively correlated with FBG and HbA1c.
Sheptulina et al. (80)	67	58 (51–65)	33.2 (30.4–36.9)	Participants with MASLD and hypertension, F and M	Cross-sectional, single-centre study	ELISA (in serum)		⁃ Participants with detectable myonectin had higher systolic blood pressure and uric acid levels. Many participants were reported to have myonectin values below the detection limit of the ELISA.

ACS, acute coronary syndrome; BMI, body mass index; CAD, coronary artery disease; Con, control; CTRP7, complement C1q tumor necrosis factor-related protein 7; DR, diabetic retinopathy; EHC, euglycemic-hyperinsulinemic clamp; ELISA, enzyme-linked immunosorbent assay; F, female; FBG, fasting blood glucose; FFA, free fatty acids; HbA1c, glycated hemoglobin A1c; HDL-C, high-density lipoprotein cholesterol; HOMA-IR, homeostatic model assessment of insulin resistance; IGT, impaired glucose tolerance; IR, insulin resistance; LSG, laparoscopic sleeve gastrectomy; M, male; MASLD, metabolic dysfunction-associated steatotic liver disease; MS, metabolic syndrome; NGT, normal glucose tolerance; NMS, non-metabolic syndrome; NPDR, non-proliferative diabetic retinopathy; nT2D, newly diagnosed type 2 diabetes; OGTT, oral glucose tolerance test; PDR, proliferative diabetic retinopathy; T2D, type 2 diabetes; TC, total cholesterol; TG, triglycerides; VO_2max_, maximal oxygen uptake.

**Table 4:** Results of randomized controlled trials of myonectin in human models.

Reference	*n*	Age (y)	BMI (kg/m^2^)	Population characteristics	Study design	Myonectin measurement technique	Intervention	Main findings
Joy al (81).	25	28 ± 5		Healthy, resistance-trained M	Randomized, double-blind, placebo-controlled	ELISA (in serum)	12 wk of periodized RT (3 d/wk) with a daily supplement of ancient peat and apple extract (150 mg)	⁃ Serum myonectin levels did not show significant changes between the treatment and placebo group.
Pourranjbar et al. (28)	80	35 – 44	>25	Obese F	RCT with control and intervention groups	ELISA (in serum)	Endurance exercise, 30 min at 50-70% HR_max_, 8 wk (3 d/wk)	⁃ Increase in serum myonectin levels and decrease in IR in the intervention group.
Sabouri et al. (82)	18	28 ± 5	Concentric: 24.12 ± 1.58 Eccentric: 22.57 ± 1.58	Healthy M	RCT with two intervention groups	RT-PCR (in vastus lateralis muscle)	12 sets of 10 reps of eccentric or concentric knee extension exercises at 60°/s.	⁃ Myonectin mRNA increased in both the eccentric and concentric groups. ⁃ No significant differences between groups.
Rezaeimaneset (83)	24	19-26	HIIT 27.3 ± 2.0 Control 27.3 ± 2.6	Overweight M	RCT with control and intervention groups	ELISA (serum)	8 wk of HIIT swimming training (3 d/wk)	⁃ The intervention increased myonectin and high-density lipoprotein levels, and decreased BMI, triglycerides, and total cholesterol.
Bahremand et al. (29)	30	20 - 40	<25	Healthy and physically active F	RCT with two intervention groups	ELISA (in serum)	Concurrent training: MICT, 60 – 80% HR_max_ for 22.5 min and RT, 65% - 80% 1-RM (progressive). CrossFit: typical WOD of this training system, 8 wk (3 d/wk)	⁃ Myonectin levels and IR index did not change in the CrossFit and concurrent training groups.
Petro et al. (84)	60	50.8 ± 6.0	30.6 ± 4.0	Obese individuals with MS, M and F	*Post-hoc* analysis of an RCT with two intervention groups	ELISA (serum)	HIIT: 90% of VO_2peak_ for a total of 22 min; 12-wk (3 d/wk) MICT: 60% of VO_2peak_ for 36 min; 12-wk (3 d/wk)	⁃ HIIT, but not MICT, significantly increased myonectin; both interventions reduced appendicular fat mass index and increased appendicular lean mass percentage.

1-RM, one-repetition maximum; BMI, body mass index; d/wk, days per week; ELISA, enzyme-linked immunosorbent assay; F, female; HDL-C, high-density lipoprotein cholesterol; HIIT, high-intensity interval training; HRmax, maximal heart rate; IR, insulin resistance; M, male; MICT, moderate-intensity continuous training; MS, metabolic syndrome; RCT, randomized controlled trial; reps, repetitions; RT, resistance training; RT-PCR, reverse transcription polymerase chain reaction; TC, total cholesterol; TG, triglycerides; VO_2peak_, peak oxygen uptake; wk, week(s); WOD, workout of the day.

## 4 Discussion

We analyzed the evidence available on the relationship between myonectin and metabolism using a comprehensive approach: bioinformatics as well as cellular and animal models provide information on the regulators, effects and mechanisms of action of myonectin with a strong causal relationship, while human studies explore how this myokine is related to clinical outcomes relevant to metabolism, although with less power on causal relationships. The main findings are the following: i) although the primary sequence of the protein is well known for several species, reliable secondary and tertiary structures are lacking; ii) myonectin is a myokine primarily expressed in skeletal muscle and is induced by long-term exercise (mainly HIIT), nutrient availability, and some exogenous compounds; iii) myonectin regulates the uptake and distribution of lipids, has potential effects on mitochondria and reduces inflammation; iv) the mechanisms of action of myonectin may be mediated by signaling pathways involving the AMP-activated protein kinase (AMPK), but also cyclic adenosine monophosphate (cAMP) and AKT; v) chronic metabolic alterations caused by obesity generally result in decreased levels of myonectin; vi) myonectin correlates negatively with alterations in lipid metabolism and abdominal fat storage.

### 4.1 Regulators of the expression of myonectin

*Fiber types and exercise:* Myonectin is primarily expressed in skeletal muscles, especially in those with a predominance of oxidative fibers (40, 55). Long-term aerobic exercise increases the expression of myonectin in skeletal muscle (in both slow and fast contraction muscle fibers) and in serum (40, 58, 63). Similarly, epinephrine, a sympathomimetic hormone; ionomycin, a Ca^2+^ ionophore that facilitates Ca^2+^ entry; and forskolin, a compound which increases cAMP, also promote the expression of myonectin in C2C12 myotubes (20). Thus, myonectin expression seems to be upregulated in response to cAMP-activating ligands and also to muscle stimuli which increase cytosolic free Ca^2+^ concentrations. Both conditions are met, for instance, during exercise. Exercise increases the levels of circulating epinephrine, in turn augmenting cAMP and Ca^2+^ release in the muscle fiber through the activation of β_2_-adrenergic receptors (85). Moreover, the repetitive muscle contraction during exercise intensely mobilizes Ca^2+^ from the intramuscular stores to the sarcoplasm (86). Interestingly, the expression of other myokines (87–89) and proteins involved in metabolism (90–92) is induced by increases in intramuscular Ca^2+^.

In humans, evidence shows that in populations without metabolic risk factors, no significant changes in myonectin concentrations are observed in response to either acute or chronic exercise (29, 66, 81). However, serum myonectin levels increased in obese individuals after eight weeks of MICT or HIIT (28, 83, 84). While the response to MICT seems to be lower and slower, HIIT is a stronger stimulus for increasing serum levels of myonectin, likely because of the more vigorous activation of the muscle tissue (84). Although the paper by Otaka et al. (23) causally linked myonectin with beneficial exercise-induced outcomes in mice, there is no evidence connecting the increase of myonectin with the exercise-favored metabolic adaptations in humans. RCT with approaches of mediation analysis may help understand if (and how much) myonectin contributes to the restoration of a healthier metabolism in response to chronic exercise (93).

It is difficult to reconcile the increases in myonectin reported in physiological and RCT studies with the lack of significant changes in the human gene expression analyses. However, although not significant, the increases in gene expression under HIIT conditions are of larger magnitude than those in MICT studies, paralleling findings in humans. The small changes support the hypothesis that the response of myonectin is low and slow, but still measurable and physiologically relevant. In any case, gene expression data is underpowered, as suggested by its large dispersion. As such, more studies are needed to clarify this dichotomy.

*Caloric and nutrient intake:* The availability of substrates (*e.g*., lipids, glucose, and amino acids) increases the expression of myonectin in skeletal muscle (20, 40). Conversely, in states of lipid disruption (*e.g.*, palmitic acid treatment in myotubes, HFD-induced obesity in animals or MS in humans), muscle dysfunction due to atrophy or aging, and starvation (*i.e*., limited substrate availability), a decrease in the expression and circulation of this myokine has been observed (20, 21, 24, 40, 44).

*Endogenous regulators: G*iven the well-recognized crosstalk between tissues through the action of humoral factors (94, 95), it is possible that many other endogenous soluble molecules (*i.e.*, besides epinephrine), such as cytokines, adipokines, myokines (including myonectin itself), FFA and miRNA, influence the expression of myonectin. Although this systematic review found limited evidence directly addressing this hypothesis, *in vitro* findings provided preliminary support. For instance, a study in murine C2C12 myotubes showed that both leptin and irisin independently increase myonectin mRNA expression, and that leptin enhances the stimulatory effect of irisin on its transcription (45). In parallel, the plausibility of this rationale is stressed by our predictive bioinformatics analysis, which highlighted hsa-miR-4251 as a potential regulator of *ERFE* expression. This miRNA has only been empirically explored as a potential regulator of histaminergic systems in breast cancer (96). Since this route has never been associated with the mechanism of action of myonectin, the potential role of hsa-miR-4251, and the other miRNAs identified, in the myonectin-metabolism relationship must be experimentally validated.

*Exogenous regulators:* Although there has not been a large screening of exogenous compounds that regulate myonectin, murine studies have shown that supplementation with vitamin B6, an essential vitamin, and the immunomodulatory polyphenol curcumin both increase its expression in skeletal muscle (54, 62). The mechanisms of this effect require clarification. Also, we recently showed that the use of statins does not appear to affect the relationship between myonectin and outcomes of body composition (21). The field is awaiting the evaluation of the effect of many active principles and drugs chronically used to treat cardiometabolic disturbances (*e.g.*, β-blockers, metformin, *etc.*) on the regulation of myonectin. This will help better understand the potential effect of these molecules as moderators in the studies and extend the knowledge on their mechanisms of action.

### 4.2 Molecular structure, effects and mechanisms of action of myonectin

*Structure and ligand-receptor binding:* The paucity of data surrounding a *bona fide* myonectin receptor, coupled with the lack of any experimental determination of the tridimensional structure of myonectin and the low quality of the predicted structures, deduced from their low confidence scores, hampers any ligand-receptor binding analysis. This limitation, in turn, hinders a deeper mechanistic understanding of the downstream activation of any intracellular signaling pathway. Moreover, with no information available to support the notion that a family of receptor isoforms might exist, as is seen for other hormones, it is difficult to disentangle the disparate effects of this myokine reported in different studies. Similarly, the study of the structural details and potential differential effects of the myonectin complexes (*i.e.*, oligomers or heteromers) (20, 41) has been limited. The use of X-ray crystallography or nuclear magnetic resonance spectroscopy techniques may help overcome this limitation in the field. In this regard, it would be of particular importance to determine with confidence the structure of the loops as well as the more disordered parts of myonectin, as these are the less precisely determined moieties in bioinformatics-predicted myonectin structures. Given their location and abundance, they are likely relevant for myonectin binding to its receptor with high specificity.

*Biological effects:* Functional studies indicate that myonectin increases the uptake of FFA in adipocytes and hepatocytes, through a mechanism mediated by an increase in the gene expression of their transporters (20, 22). This increase in FFA availability in the cytoplasm could, depending on the conditions and cell type, lead to greater fat accumulation or promote their oxidation. In this context, it has been demonstrated that myonectin has anti-adipogenic effects, induces the expression of AMPK, improves mitochondrial function markers and favors a lipolytic environment (22, 48). Overall, this is likely to aid in controlling the healthy expansion of adipose tissue and improving global metabolic health.

In scenarios where myonectin improves metabolism and adipocyte health, an anti-inflammatory effect would be expected. To date, there is a few evidence to support this. For instance, myonectin significantly downregulates the expression of TNF-α, IL-6 (23), and MCP-1, and the oxygen consumption rate (OCR) in RAW264.7 immune cells (47), suggesting an immunomodulatory effect. However, comprehensive evaluations of how myonectin influences the global and localized inflammatory profile in several tissues are lacking.

If myonectin has a measurable role in lipid metabolism and inflammation, it is expected to have a role in the pathophysiology of chronic metabolic conditions, then a relationship between myonectin and relevant clinical metabolic outcomes in humans under different contexts can be anticipated. However, despite consistency across several preclinical studies, translation of findings from cells and animals to humans has not been straightforward. While some studies report elevated levels of myonectin in patients with these conditions (25, 26, 65, 72), others report decreased levels compared to control individuals (21, 27). Similarly, observed relationships between myonectin and serum FFA have not been consistent across studies.

These discrepancies could be explained by multiple factors, including:

- The heterogeneity of the studied populations: Multiple characteristics could influence myonectin levels, including age, ethnicity, body composition (*e.g.*, fat distribution and muscle mass), physical fitness, IR, disease progression and comorbidities, iron levels, and use of medications. It is also plausible that variations in myonectin levels are associated with different metabolic disease phenotypes. For instance, obese individuals with excess visceral fat, lower muscle mass, and lower physical fitness may have lower myonectin levels compared to those who do not exhibit these characteristics. Additionally, it is important to consider the temporal course of the disease (*e.g*., newly diagnosed individuals versus those in advanced stages), as high myonectin levels in some studies could reflect compensatory responses to cardiometabolic alterations, while lower levels of myonectin may be observed as individuals experience greater metabolic alterations and decline in muscle status (*e.g.*, sarcopenic, with high myosteatosis).
- Unsystematic adjustment for confounders: The pleiotropic role of myonectin as a regulator of iron homeostasis is relevant as an example in this case. Myonectin interferes with hepcidin transcription and promotes greater iron absorption and mobilization from stores (97, 98). Since individuals with MS may present with iron overload and elevated serum ferritin and hepcidin levels (99), it is possible that reduced myonectin concentrations could act as a feedback mechanism to manage this overload. Unfortunately, iron status has not been adjusted for in the epidemiological studies.
- Differences in measurement methods: The wide range of blood myonectin concentrations (~0.1–400 ng/mL) reported in humans are indicative of large technical and methodological differences across the studies. This variability can be partly attributed to the use of various enzyme-linked immunosorbent assay (ELISA) kits to analyze myonectin levels, limiting the comparability of results between different investigations. Furthermore, differences in sample processing, such as analysis in serum or plasma, might also contribute to the observed disparity in myonectin levels among studies.
- Lack of feasibility in directly translating results from animals to humans: The concentrations of myonectin employed in experiments with cellular and murine models (~0.5–10 μg/mL) are at least three orders of magnitude higher than those found in circulation in humans. Thus, it is possible that not all the effects of myonectin observed in the preclinical models can be reproduced under physiological conditions in humans. Future experiments of basic sciences should be performed with more physiological (murine and human) concentrations of myonectin and the sequences of the recombinant peptides utilized should be clearly reported.

Considering the articles with the most robust evidence and lower risk of bias, as given by the larger and more homogeneous samples, use of controls, high precision in the measurements, adjustments for confounders, and the most complete and detailed reporting of results, it is possible to propose that myonectin tends to be reduced in presence of moderate and severe metabolic disturbances and is inversely related to markers of abdominal obesity, confirming its role in controlling the distribution and expansion of the adipose tissue. The relationship observed in mice between myonectin and serum FFA, however, has definitively not been reproduced in humans.

*Mechanisms of action:* Despite the above-mentioned limitations in the understanding of the ligand-receptor interactions, a study revealed that a mutant version of myonectin could weakly interact with the β_2_ adrenergic receptor, whose intracellular pathway is mediated by cAMP. Mutant myonectin was also shown to form a more stable complex with the insulin receptor, a tyrosine kinase receptor well known to downstream activate AKT. As expected, this genetic modification, delivered via an optimized AAV vector, improved glucose tolerance and IR in obese mice (61).

In agreement with the beneficial profile driven by the activation of cAMP/AKT mediated routes, myonectin has shown protective and survival effects in other cell types under specific pathological conditions. For instance, in a model of cardiac hypoxia-reoxygenation, myonectin reduced cardiomyocyte apoptosis and ameliorated the inflammatory response of macrophages stimulated with lipopolysaccharides through the activation of the S1P-dependent cAMP/AKT pathway, thus being cardioprotective (23). Myonectin also inhibited autophagy in hepatocytes by suppressing the formation of autophagosomes in a cAMP/AKT-dependent manner (40).

Myonectin seems also to stimulate the AMPK pathway, which is particularly relevant for explaining its beneficial role in metabolism. The AMPK pathway increases FFA and glucose oxidation as well as mitochondrial biogenesis in several metabolically active tissues, such as the liver, fat and skeletal muscle. Consistent with the autocrine effects of other myokines (100), Ozaki et al. (24) demonstrated that myonectin preserves muscular structural parameters and mitochondrial markers (*e.g.*, number, structure and expression of Tfam, Sirt1, and Nrf), possibly through the activation of the AMPK/PGC1-alpha pathway (24).

Myonectin belongs to the CTPR family of proteins (20, 41), which have affinity for adiponectin receptors (AdipoR1, AdipoR2 and Cadherin) (101). Given that AdipoR are present in several metabolically relevant tissues and activate downstream pathways such as AMPK (102), it is possible that the primary route activated by myonectin is mediated by AMPK. The stimulation of AdipoR and AMPK-mediated routes by myonectin would be consistent with many of the observed metabolic effects of this myokine: improving glycemic control, increasing fatty acid oxidation and exerting anti-inflammatory effects (103).

In this context, since myonectin induces the expression of FFA transporters and activates AMPK, it would be expected that the transported FFA are not mainly stored, but oxidated. It is noteworthy that the effect of myonectin on the OCR, an indicator of mitochondrial function and substrates oxidation, has not yet been evaluated in skeletal muscle cells or adipocytes. Such studies would provide insights into weather myonectin favors the oxidation of substrates. As myonectin reduces OCR in RAW264.7 immune cells, but does not affect OCR in mouse osteoblasts (47), it is difficult to conclude on a general effect of myonectin on OCR in different tissues, especially because immune cells and osteoblasts are not particularly relevant to the lipid metabolism and the global metabolic health.

**Figure 8:**
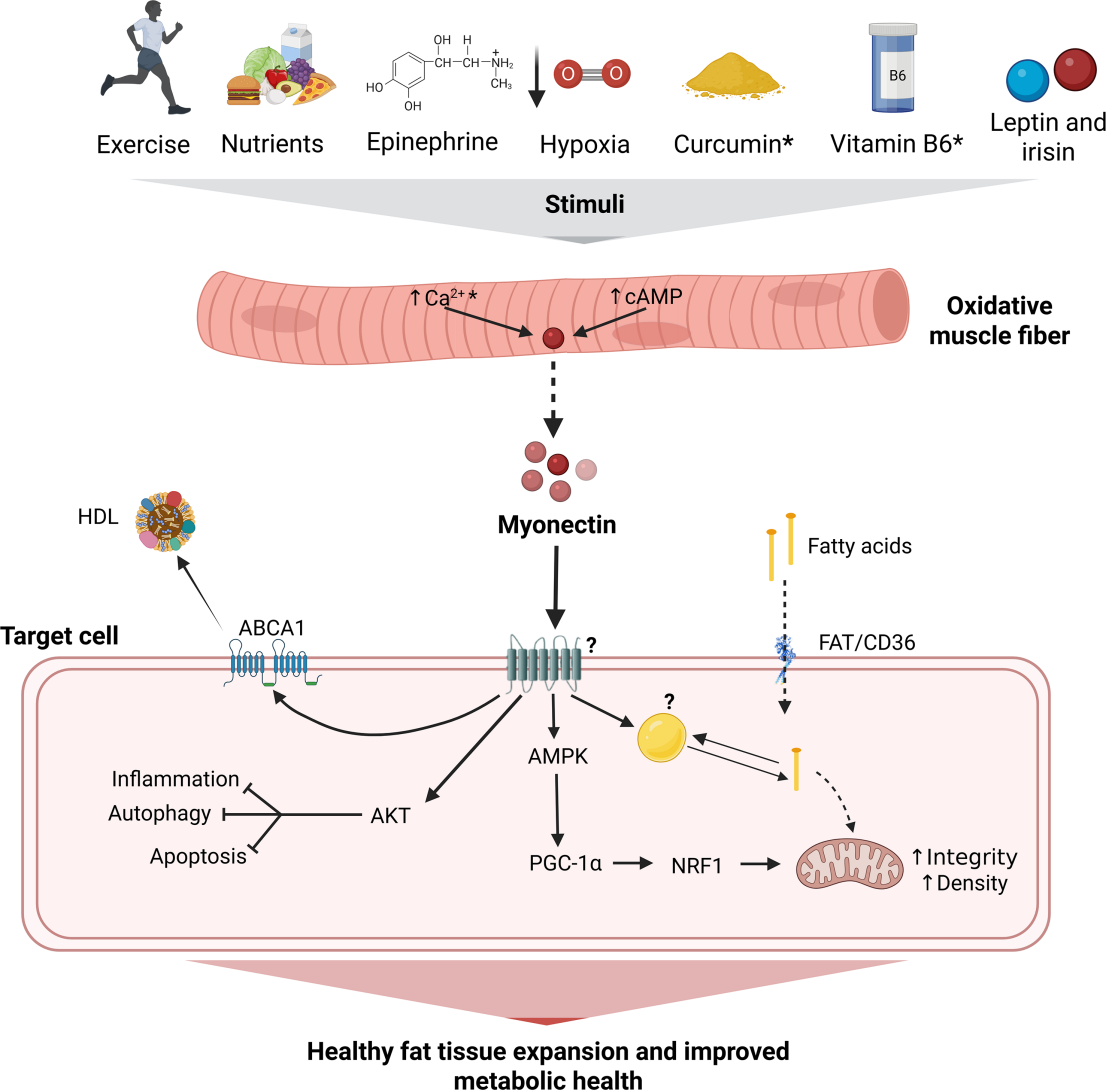
Integrative model of the effects of myonectin on metabolic health. Myonectin is a myokine secreted primarily by oxidative muscle fibers in response to factors such as exercise, nutrient intake (*e.g*., glucose, amino acids, fatty acids), epinephrine and curcumin, possibly mediated by an increase in cytoplasmic levels of cAMP and Ca^2+^. Once released, myonectin acts through an as-yet-unidentified receptor, modulating key pathways, such as those related to AMPK and AKT, in different cell types. This regulation includes fatty acid transport via FAT/CD36, the promotion of mitochondrial integrity and density through PGC-1α and NRF1, and the inhibition of processes such as inflammation, autophagy, and apoptosis. Additionally, it increases ABCA1 expression, promoting cholesterol efflux to HDL. Taken together, these mechanisms contribute to healthy adipose tissue expansion and improved metabolic health. Under chronic metabolic alterations caused by obesity myonectin becomes reduced, thus limiting its beneficial effects. ABCA1, ATP-binding cassette transporter A1; AKT, serine/threonine-protein kinase Akt; AMPc, cyclic adenosine monophosphate; AMPK, AMP-activated protein kinase; Ca²^+^, calcium; CD36/FAT, cluster of differentiation 36/fatty acid translocase; HDL, high-density lipoprotein; myonectin; NRF1, nuclear respiratory factor 1; PGC-1α, peroxisome proliferator-activated receptor gamma coactivator 1-alpha. **→** indicate stimulation; **⊣** inhibition; ↑ increase; * they must be confirmed by additional studies (*i.e*., the available evidence comes from a single study and/or the underlying mechanisms need to be evaluated); ? effect is not completely clear or is unknown. Created in BioRender.

One final interesting issue is the fact that myonectin seems to activate signaling pathways that may have opposing functions. While AKT inhibits autophagy but increases fatty acid, glycogen and protein synthesis, AMPK does the opposite. Activating AKT and AMPK is not necessarily incompatible; during exercise, both pathways may be engaged in a complementary or temporally coordinated manner, leading to additive effects that increase glucose uptake by the skeletal muscle (104). Finding the primary receptor of myonectin and assessing the impact of myonectin on the signaling pathways and mitochondrial function in cells that play a central role in metabolism is pertinent to determine what metabolic cellular phenotype myonectin really contributes to, either under health or diseased conditions. **Figure 8** shows our proposed model of the effects of myonectin on metabolic health.

### 4.3 Strengths and limitations

Even though we performed a systematic review of the literature spanning molecules, cells, animals and humans, and complemented this with bioinformatic and bibliometric tools to have a broader and deeper landscape of the field, Chinese and African databases were not searched. Accordingly, we acknowledge that some papers may have been missed, particularly if they were not in available in English. Also, considering that we did not present a meta-analysis, we were not interested in estimating a common effect. Therefore, a publication bias assessment as usually shown in funnel plots or Egger tests was not carried out. Overall, our conclusions might not be generalizable worldwide.

Albeit several papers did not completely adhere to the expected reporting of results, thus raising concerns about a moderate-high risk of bias, they were otherwise critically judged as being apparently free of other problems that could result in high risk of bias in the quality of their science. These studies were thus included in the review.

Naming myonectin as its synonym complement C1q tumor necrosis factor-related protein 15 (CTRP15) has led some groups to a confusion with the complement C1q tumor necrosis factor-related protein 5 (CTRP5, UniProtKB Q9BXJ0, gene C1QTNF5) (25). CTRP15 and CTRP5 are two distinct proteins that share similarities in their C1q domains but exhibit differences in their sequences and specific functions (101). To avoid misunderstandings, it is recommended to use the names as registered in specialized repositories or databases, as we did in this review, thus ensuring that all information included was related specifically to myonectin.

### 4.4 Future research directions

Identification of the myonectin receptor and increasing knowledge about the structure of myonectin will allow for biochemical and biophysical studies on ligand-receptor interaction to be performed. Also, implementing physiological approaches in living cells to further investigate aspects such as mitochondrial density and dynamics, mitochondrial potential, and oxidative stress, as well as exploring the crosstalk between myonectin and other myokines, cytokines of adipokines, are needed to improve understanding of the effects of myonectin on metabolism and its contribution to the pathophysiology of chronic metabolic diseases. This could be achieved through approaches such as fluorescence microscopy and metabolic flux analysis, focusing on closely mimicking the physiological milieu of cells. Finally, transcriptomic and proteomic studies that delineate the molecular pathways that are regulated by myonectin will complement the physiological studies with information about the mechanisms of action.

In humans, high-quality RCT utilizing various exercise protocols (*e.g.*, MICT, HIIT, and resistance training) and loads are required to assess how different exercise paradigms affect the levels of myonectin inside the skeletal muscles and in serum. In addition to conventional techniques for evaluating clinical outcomes, the implementation of methods that simultaneously measure multiple secreted factors (105), and the improved standardization of ELISA or Multiplex protocols that avoid the need to dilute the samples will reduce variability among studies. The use of non-invasive assessment of body composition and myosteatosis (*e.g.*, dual-energy X-ray absorptiometry, computed tomography, magnetic resonance for imaging and spectroscopy) (84, 106), moving beyond the estimation of the BMI, with designs of causal mediation analysis (93), and a proper adjustment for relevant confounders (*i.e.*, sex, age, lean mass, fat mass, physical activity, iron status, medications) will enhance our understanding of the secretory function of the muscle in the context of metabolic disorders with more reliability. Finally, since the responses of myonectin to different stimuli seem to be low and slow, and thus technically and methodologically challenging, studies should allocate larger samples with improved randomization protocols, avoid co-interventions, ensure a minimum follow-up of 16 weeks and appropriately handle missing data.

## 5 Conclusions

Given its positive role in lipid uptake, the likely AMPK and mitochondrial activation, its negative relationship with abdominal obesity and its upregulation with exercise, and its immunomodulatory effect, myonectin appears to favor the oxidation of fat instead of only its storage, thus boosting a healthy expansion and distribution of the adipose tissue. The reduction in myonectin seen in common chronic metabolic diseases may thus favor the unhealthy expansion of adipose tissue, driving the well-known associated detrimental effects on the metabolic health.

Moving forward, research that prioritizes the understanding of the signaling pathways activated by myonectin will enhance our understanding of its function and involvement in the pathophysiology of chronic metabolic diseases.

## Data Availability

The datasets presented in this article are not readily available because the data used in this review were obtained from public databases and have no access restrictions. The bioinformatic analysis was conducted using data available on platforms such as UniProt and AlphaFold, which can be directly accessed through the specific identifiers provided in the article. The authors are available to provide additional information upon request. Requests to access the datasets should be directed to Jorge Luis Petro, jorgelpetro@correo.unicordoba.edu.co.

## References

[1] Green H Goreham C Ouyang J Ball-Burnett M Ranney D . Regulation of fiber size, oxidative potential, and capillarization in human muscle by resistance exercise. Am J Physiol. (1999) 276:R591–6. doi: 10.1152/ajpregu.1999.276.2.R591, PMID: 9950941

[2] Luden N Hayes E Minchev K Louis E Raue U Conley T . Skeletal muscle plasticity with marathon training in novice runners. Scand J Med Sci Sports. (2012) 22:662–70. doi: 10.1111/j.1600-0838.2011.01305.x, PMID: 21477203

[3] Deshmukh AS Steenberg DE Hostrup M Birk JB Larsen JK Santos A . Deep muscle-proteomic analysis of freeze-dried human muscle biopsies reveals fiber type-specific adaptations to exercise training. Nat Commun. (2021) 12:304. doi: 10.1038/s41467-020-20556-8, PMID: 33436631 PMC7803955

[4] Du J Yun H Wang H Bai X Su Y Ge X . Proteomic profiling of muscular adaptations to short-term concentric versus eccentric exercise training in humans. Mol Cell Proteomics. (2024) 23:100748. doi: 10.1016/j.mcpro.2024.100748, PMID: 38493954 PMC11017286

[5] DeFronzo RA Jacot E Jequier E Maeder E Wahren J Felber JP . The effect of insulin on the disposal of intravenous glucose: results from indirect calorimetry and hepatic and femoral venous catheterization. Diabetes. (1981) 30:1000–7. doi: 10.2337/diab.30.12.1000, PMID: 7030826

[6] Ibrahimi A Bonen A Blinn WD Hajri T Li X Zhong K . Muscle-specific overexpression of FAT/CD36 enhances fatty acid oxidation by contracting muscle, reduces plasma triglycerides and fatty acids, and increases plasma glucose and insulin. J Biol Chem. (1999) 274:26761–6. doi: 10.1074/jbc.274.38.26761, PMID: 10480880

[7] Rowe GC El-Khoury R Patten IS Rustin P Arany Z . PGC1α dispensable for exercise-induced mitochondrial biogenesis in skeletal muscle. PloS One. (2012) 7:e41817. doi: 10.1371/journal.pone.0041817, PMID: 22848618 PMC3404101

[8] Gallo-Villegas JA Calderón JC . Epidemiological, mechanistic, and practical bases for assessment of cardiorespiratory fitness and muscle status in adults in healthcare settings. Eur J Appl Physiol. (2023) 123:945–64. doi: 10.1007/s00421-022-05114-y, PMID: 36683091 PMC10119074

[9] Little HC Tan SY Cali FM Rodriguez S Lei X Wolfe A . Multiplex quantification identifies novel exercise-regulated myokines/cytokines in plasma and in glycolytic and oxidative skeletal muscle. Mol Cell Proteomics. (2018) 17:1546–63. doi: 10.1074/mcp.RA118.000794, PMID: 29735541 PMC6072542

[10] Boström P Wu J Jedrychowski MP Korde A Ye L Lo JC . A PGC1α dependent myokine that drives brown-fat-like development of white fat and thermogenesis. Nature. (2012) 481:463–8. doi: 10.1038/nature10777, PMID: 22237023 PMC3522098

[11] Vinel C Lukjanenko L Batut A Deleruyelle S Pradere JP Le Gonidec S . The exerkine apelin reverses age-associated sarcopenia. Nat Med. (2018) 24:1360–71. doi: 10.1038/s41591-018-0131-6, PMID: 30061698

[12] Murillo-Saich JD Vazquez-Villegas ML Ramirez-Villafaña M Saldaña-Cruz AM Aceves-Aceves JA Gonzalez-Lopez L . Association of myostatin, a cytokine released by muscle, with inflammation in rheumatoid arthritis: A cross-sectional study. Med (Baltimore). (2021) 100:e24186. doi: 10.1097/md.0000000000024186, PMID: 33546034 PMC7837870

[13] Zhu H Liu D Sui M Zhou M Wang B Qi Q . CRISPRa-based activation of Fgf21 and Fndc5 ameliorates obesity by promoting adipocytes browning. Clin Transl Med. (2023) 13:e1326. doi: 10.1002/ctm2.1326, PMID: 37462619 PMC10353577

[14] Narvaez-Sanchez R Calderón JC Vega G Trillos MC Ospina S . Skeletal muscle as a protagonist in the pregnancy metabolic syndrome. Med Hypotheses. (2019) 126:26–37. doi: 10.1016/j.mehy.2019.02.049, PMID: 31010495

[15] Norheim F Raastad T Thiede B Rustan AC Drevon CA Haugen F . Proteomic identification of secreted proteins from human skeletal muscle cells and expression in response to strength training. Am J Physiol Endocrinol Metab. (2011) 301:E1013–21. doi: 10.1152/ajpendo.00326.2011, PMID: 21828336

[16] Henningsen J Rigbolt KT Blagoev B Pedersen BK Kratchmarova I . Dynamics of the skeletal muscle secretome during myoblast differentiation. Mol Cell Proteomics. (2010) 9:2482–96. doi: 10.1074/mcp.M110.002113, PMID: 20631206 PMC2984231

[17] Hartwig S Raschke S Knebel B Scheler M Irmler M Passlack W . Secretome profiling of primary human skeletal muscle cells. Biochim Biophys Acta. (2014) 1844:1011–7. doi: 10.1016/j.bbapap.2013.08.004, PMID: 23994228

[18] Sánchez YL Yepes-Calderón M Valbuena L Milán AF Trillos-Almanza MC Granados S . Musclin is related to insulin resistance and body composition, but not to body mass index or cardiorespiratory capacity in adults. Endocrinol Metab (Seoul). (2021) 36:1055–68. doi: 10.3803/EnM.2021.1104, PMID: 34674511 PMC8566119

[19] Fu S Xing G . Changes in serum irisin levels and their significance in carotid atherosclerosis associated with obesity. Altern Ther Health Med. (2024) 30(12):194–99., PMID: 38518138

[20] Seldin MM Peterson JM Byerly MS Wei Z Wong GW . Myonectin (CTRP15), a novel myokine that links skeletal muscle to systemic lipid homeostasis. J Biol Chem. (2012) 287:11968–80. doi: 10.1074/jbc.M111.336834, PMID: 22351773 PMC3320944

[21] Petro JL Fragozo-Ramos MC Milán AF Aristizabal JC Gallo-Villegas JA Calderón JC . Serum levels of myonectin are lower in adults with metabolic syndrome and are negatively correlated with android fat mass. Int J Mol Sci. (2023) 24:6874. doi: 10.3390/ijms24086874, PMID: 37108038 PMC10138930

[22] Sun Z Liu Z Xi J Liu Y Zheng Z Li N . Effects of myonectin on porcine intramuscular adipocyte differentiation and exogenous free fatty acid utilization. Anim Biotechnol. (2023) 34:3757–64. doi: 10.1080/10495398.2023.2224838, PMID: 37382421 PMC13353430

[23] Otaka N Shibata R Ohashi K Uemura Y Kambara T Enomoto T . Myonectin is an exercise-induced myokine that protects the heart from ischemia-reperfusion injury. Circ Res. (2018) 123:1326–38. doi: 10.1161/circresaha.118.313777, PMID: 30566056

[24] Ozaki Y Ohashi K Otaka N Kawanishi H Takikawa T Fang L . Myonectin protects against skeletal muscle dysfunction in male mice through activation of AMPK/PGC1α pathway. Nat Commun. (2023) 14:4675. doi: 10.1038/s41467-023-40435-2, PMID: 37542026 PMC10403505

[25] Li K Liao X Wang K Mi Q Zhang T Jia Y . Myonectin predicts the development of type 2 diabetes. J Clin Endocrinol Metab. (2018) 103:139–47. doi: 10.1210/jc.2017-01604, PMID: 29161407

[26] Mi Q Li Y Wang M Yang G Zhao X Liu H . Circulating C1q/TNF-related protein isoform 15 is a marker for the presence of metabolic syndrome. Diabetes Metab Res Rev. (2019) 35:e3085. doi: 10.1002/dmrr.3085, PMID: 30303269

[27] Li Z Yang YL Zhu YJ Li CG Tang YZ Ni CL . Circulating serum myonectin levels in obesity and type 2 diabetes mellitus. Exp Clin Endocrinol Diabetes. (2021) 129:528–34. doi: 10.1055/a-0896-8548, PMID: 31340393

[28] Pourranjbar M Arabnejad N Naderipour K Rafie F . Effects of aerobic exercises on serum levels of myonectin and insulin resistance in obese and overweight women. J Med Life. (2018) 11:381–6. doi: 10.25122/jml-2018-0033, PMID: 30894898 PMC6418335

[29] Bahremand M Hakak Dokht E Moazzami M . A comparison of crossfit and concurrent training on myonectin, insulin resistance and physical performance in healthy young women. Arch Physiol Biochem. (2023) 129:603–9. doi: 10.1080/13813455.2020.1853173, PMID: 33259247

[30] UniProt Consortium . Uniprot: the universal protein knowledgebase in 2023. Nucleic Acids Res. (2023) 51:D523–d31. doi: 10.1093/nar/gkac1052, PMID: 36408920 PMC9825514

[31] Wu S Skolnick J Zhang Y . Ab initio modeling of small proteins by iterative tasser simulations. BMC Biol. (2007) 5:17. doi: 10.1186/1741-7007-5-17, PMID: 17488521 PMC1878469

[32] Jumper J Evans R Pritzel A Green T Figurnov M Ronneberger O . Highly accurate protein structure prediction with alphafold. Nature. (2021) 596:583–9. doi: 10.1038/s41586-021-03819-2, PMID: 34265844 PMC8371605

[33] Varadi M Bertoni D Magana P Paramval U Pidruchna I Radhakrishnan M . Alphafold protein structure database in 2024: providing structure coverage for over 214 million protein sequences. Nucleic Acids Res. (2024) 52:D368–D75. doi: 10.1093/nar/gkad1011, PMID: 37933859 PMC10767828

[34] Waterhouse A Bertoni M Bienert S Studer G Tauriello G Gumienny R . Swiss-model: homology modelling of protein structures and complexes. Nucleic Acids Res. (2018) 46:W296–w303. doi: 10.1093/nar/gky427, PMID: 29788355 PMC6030848

[35] Pillon NJ Gabriel BM Dollet L Smith JAB Sardón Puig L Botella J . Transcriptomic profiling of skeletal muscle adaptations to exercise and inactivity. Nat Commun. (2020) 11:470. doi: 10.1038/s41467-019-13869-w, PMID: 31980607 PMC6981202

[36] Marzi G Balzano M Caputo A Pellegrini MM . Guidelines for bibliometric-systematic literature reviews: 10 steps to combine analysis, synthesis and theory development. Int J Manag Rev. (2024) 27:81–103. doi: 10.1111/ijmr.12381

[37] Page MJ McKenzie JE Bossuyt PM Boutron I Hoffmann TC Mulrow CD . The prisma 2020 statement: an updated guideline for reporting systematic reviews. BMJ. (2021) 372:n71. doi: 10.1136/bmj.n71, PMID: 33782057 PMC8005924

[38] Hooijmans CR Rovers MM de Vries RB Leenaars M Ritskes-Hoitinga M Langendam MW . Syrcle’s risk of bias tool for animal studies. BMC Med Res Methodol. (2014) 14:43. doi: 10.1186/1471-2288-14-43, PMID: 24667063 PMC4230647

[39] Martimbianco ALC Sá KMM Santos GM Santos EM Pacheco RL Riera R . Most Cochrane systematic reviews and protocols did not adhere to the cochrane’s risk of bias 2.0 tool. Rev Assoc Med Bras (1992). (2023) 69:469–72. doi: 10.1590/1806-9282.20221593, PMID: 36820779 PMC10004297

[40] Seldin MM Lei X Tan SY Stanson KP Wei Z Wong GW . Skeletal muscle-derived myonectin activates the mammalian target of rapamycin (mTOR) pathway to suppress autophagy in liver. J Biol Chem. (2013) 288:36073–82. doi: 10.1074/jbc.M113.500736, PMID: 24187137 PMC3861655

[41] Stewart AN Little HC Clark DJ Zhang H Wong GW . Protein modifications critical for myonectin/erythroferrone secretion and oligomer assembly. Biochemistry. (2020) 59:2684–97. doi: 10.1021/acs.biochem.0c00461, PMID: 32602701 PMC7683180

[42] Percie du Sert N Hurst V Ahluwalia A Alam S Avey MT Baker M . The arrive guidelines 2.0: updated guidelines for reporting animal research. BMJ Open Sci. (2020) 4:e100115. doi: 10.1136/bmjos-2020-100115, PMID: 34095516 PMC7610906

[43] Little HC Rodriguez S Lei X Tan SY Stewart AN Sahagun A . Myonectin deletion promotes adipose fat storage and reduces liver steatosis. FASEB J. (2019) 33:8666–87. doi: 10.1096/fj.201900520R, PMID: 31002535 PMC6593887

[44] Yang M Wei D Mo C Zhang J Wang X Han X . Saturated fatty acid palmitate-induced insulin resistance is accompanied with myotube loss and the impaired expression of health benefit myokine genes in C2C12 myotubes. Lipids Health Dis. (2013) 12:104. doi: 10.1186/1476-511x-12-104, PMID: 23866690 PMC3723881

[45] Rodriguez A Becerril S Mendez-Gimenez L Ramirez B Sainz N Catalan V . Leptin administration activates irisin-induced myogenesis via nitric oxide-dependent mechanisms, but reduces its effect on subcutaneous fat browning in mice. Int J Obes (Lond). (2015) 39:397–407. doi: 10.1038/ijo.2014.166, PMID: 25199621

[46] Zhao Q Zhang CL Xiang RL Wu LL Li L . CTRP15 derived from cardiac myocytes attenuates TGFβ1-induced fibrotic response in cardiac fibroblasts. Cardiovasc Drugs Ther. (2020) 34:591–604. doi: 10.1007/s10557-020-06970-6, PMID: 32424654

[47] Kawaguchi M Kawao N Takafuji Y Ishida M Kaji H . Myonectin inhibits the differentiation of osteoblasts and osteoclasts in mouse cells. Heliyon. (2020) 6:e03967. doi: 10.1016/j.heliyon.2020.e03967, PMID: 32514479 PMC7266783

[48] Park TJ Park A Kim J Kim JY Han BS Oh KJ . Myonectin inhibits adipogenesis in 3T3-L1 preadipocytes by regulating P38 MAPK pathway. BMB Rep. (2021) 54:124–9. doi: 10.5483/BMBRep.2021.54.2.262, PMID: 33407993 PMC7907746

[49] Ahmadi R Fadaei R Shokoohi Nahrkhalaji A Panahi G Fallah S . The impacts of C1q/TNF-related protein-15 and adiponectin on interleukin-6 and tumor necrosis factor-α in primary macrophages of patients with coronary artery diseases. Cytokine. (2021) 142:155470. doi: 10.1016/j.cyto.2021.155470, PMID: 33676229

[50] Takasawa S Shobatake R Itaya-Hironaka A Makino M Uchiyama T Sakuramoto-Tsuchida S . Upregulation of IL-8, osteonectin, and myonectin mRNAs by intermittent hypoxia via OCT1- and NRF2-mediated mechanisms in skeletal muscle cells. J Cell Mol Med. (2022) 26:6019–31. doi: 10.1111/jcmm.17618, PMID: 36457269 PMC9753449

[51] Sharma N Castorena CM Cartee GD . Greater insulin sensitivity in calorie restricted rats occurs with unaltered circulating levels of several important myokines and cytokines. Nutr Metab (Lond). (2012) 9:90. doi: 10.1186/1743-7075-9-90, PMID: 23067400 PMC3541154

[52] Peterson JM Mart R Bond CE . Effect of obesity and exercise on the expression of the novel myokines, myonectin and fibronectin type III domain containing 5. PeerJ. (2014) 2:e605. doi: 10.7717/peerj.605, PMID: 25289190 PMC4184026

[53] Adigozalpour M Safarzade A . Effect of resistance training with two different volumes on serum myonectin levels in rats fed with sucrose solution. Ann Appl Sport Sci. (2017) 5:11–9. doi: 10.18869/acadpub.aassjournal.5.2.11

[54] Suidasari S Uragami S Yanaka N Kato N . Dietary vitamin B6 modulates the gene expression of myokines, Nrf2-related factors, myogenin and HSP60 in the skeletal muscle of rats. Exp Ther Med. (2017) 14:3239–46. doi: 10.3892/etm.2017.4879, PMID: 28912874 PMC5585884

[55] Jia WH Wang NQ Yin L Chen X Hou BY Qiang GF . Effect of skeletal muscle phenotype and gender on fasting-induced myokine expression in mice. Biochem Biophys Res Commun. (2019) 514:407–14. doi: 10.1016/j.bbrc.2019.04.155, PMID: 31056256

[56] Koohestani Sini Z Afzalpour ME Mohammadnia Ahmadi M Sardar MA Gorgani Firuzjaee S . The effect of aerobic continuous training on myonectin, insulin resistance and liver enzymes in rats with nonalcoholic fatty liver disease. Ann Appl Sport Sci. (2020) 8(S2):e855. doi: 10.29252/aassjournal.e855

[57] Gauze-Gnagne C Raynaud F Djohan YF Lauret C Feillet-Coudray C Coudray C . Impact of diets rich in olive oil, palm oil or lard on myokine expression in rats. Food Funct. (2020) 11:9114–28. doi: 10.1039/d0fo01269f, PMID: 33025998

[58] Rahmati-Ahmadabad S Rostamkhani F Meftahi GH Shirvani H . Comparative effects of high-intensity interval training and moderate-intensity continuous training on soleus muscle fibronectin type III domain-containing protein 5, myonectin and glucose transporter type 4 gene expressions: A study on the diabetic rat model. Mol Biol Rep. (2021) 48:6123–9. doi: 10.1007/s11033-021-06633-1, PMID: 34374894

[59] Koohestani Sini Z Afzalpour ME Ahmadi MM Sardar MA Khaleghzadeh H Gorgani-Firuzjaee S . Comparison of the effects of high-intensity interval training and moderate-intensity continuous training on indices of liver and muscle tissue in high-fat diet-induced male rats with non-alcoholic fatty liver disease. Egyptian Liver J. (2022) 12(63). doi: 10.1186/s43066-022-00229-5

[60] Tan WH Peng ZL You T Sun ZL . CTRP15 promotes macrophage cholesterol efflux and attenuates atherosclerosis by increasing the expression of ABCA1. J Physiol Biochem. (2022) 78:653–66. doi: 10.1007/s13105-022-00885-6, PMID: 35286626

[61] Qi Z Xia J Xue X Liu W Huang Z Zhang X . Codon-optimized FAM132b gene therapy prevents dietary obesity by blockading adrenergic response and insulin action. Int J Obes (Lond). (2022) 46:1970–82. doi: 10.1038/s41366-022-01189-x, PMID: 35922561

[62] Hu M Han M Zhang H Li Z Xu K Kang H . Curcumin (Cuminup60^®^) mitigates exercise fatigue through regulating PI3K/Akt/AMPK/mTOR pathway in mice. Aging (Albany NY). (2023) 15:2308–20. doi: 10.18632/aging.204614, PMID: 36988546 PMC10085593

[63] Özçatal Y Akat F Tatar Y Fıçıcılar H Serdaroğlu B Topal Çelikkan F . Effects of high-intensity interval training (HIIT) on skeletal muscle atrophy, function, and myokine profile in diabetic myopathy. Cytokine. (2023) 169:156279. doi: 10.1016/j.cyto.2023.156279, PMID: 37329818

[64] Avcu EC Cinar V Yasul Y Akbulut T Pancar Z Aydemir IS . Effects of an energy drink on myonectin in the liver, kidney and skeletal muscle of exercised rats. Biotech Histochem. (2024) 99:69–75. doi: 10.1080/10520295.2024.2305113, PMID: 38293763

[65] Toloza FJK Mantilla-Rivas JO Perez-Matos MC Ricardo-Silgado ML Morales-Alvarez MC Pinzon-Cortes JA . Plasma levels of myonectin but not myostatin or fibroblast-derived growth factor 21 are associated with insulin resistance in adult humans without diabetes mellitus. Front Endocrinol. (2018) 9:5. doi: 10.3389/fendo.2018.00005, PMID: 29445355 PMC5797732

[66] Kamiński M Kippen J Gomulska A Smyrak J Karolewski M Bielawska L . Myonectin serum concentration changes after short-term physical activity among young, healthy people. Med Res J. (2019) 4:41–5. doi: 10.5603/MRJ.a2019.0002

[67] Li L Wang Q Qin C . Serum myonectin is increased after laparoscopic sleeve gastrectomy. Ann Clin Biochem. (2020) 57:360–4. doi: 10.1177/0004563220942263, PMID: 32588645

[68] Meinecke A Mitzka S Just A Cushman S Stojanovic SD Xiao K . Cardiac endurance training alters plasma profiles of circular RNA MBOAT2. Am J Physiol Heart Circ Physiol. (2020) 319:H13–21. doi: 10.1152/ajpheart.00067.2020, PMID: 32412780

[69] Zhang J Hu W Lin P Wang R . Decreased serum myonectin concentrations in diabetic nephropathy patients. Clin Exp Med. (2020) 20:601–7. doi: 10.1007/s10238-020-00654-z, PMID: 32852729

[70] Mohassel Azadi S Shateri H Mohammadi M Fadaei R Sajedi F Ziamajidi N . Increased circulating level of CTRP15 in patients with type 2 diabetes mellitus and its relation with inflammation and insulin resistance. J Diabetes Metab Disord. (2021) 20:1499–504. doi: 10.1007/s40200-021-00892-2, PMID: 34900801 PMC8630316

[71] Sun H Li Z Hu W Ma W . Association of serum and aqueous humor myonectin concentrations with diabetic retinopathy. Sci Rep. (2021) 11(1):7215. doi: 10.1038/s41598-021-86677-2, PMID: 33785845 PMC8009941

[72] Shokoohi Nahrkhalaji A Ahmadi R Fadaei R Panahi G Razzaghi M Fallah S . Higher serum level of CTRP15 in patients with coronary artery disease is associated with disease severity, body mass index and insulin resistance. Arch Physiol Biochem. (2022) 128:276–80. doi: 10.1080/13813455.2019.1675713, PMID: 31608708

[73] Liu Y Wei C Ding Z Xing E Zhao Z Shi F . Role of serum C1q/TNF-related protein family levels in patients with acute coronary syndrome. Front Cardiovasc Med. (2022) 9:967918. doi: 10.3389/fcvm.2022.967918, PMID: 36061536 PMC9437344

[74] Al-Regaiey KA Habib SS Alhaqbani AO Alhamed MS Alsalman MA Alhadlaq AA . Decreased plasma myonectin levels in female patients with type 2 diabetes mellitus and its correlation with lipid and glycemic parameters. Eur Rev Med Pharmacol Sci. (2023) 27:8773–9. doi: 10.26355/eurrev_202309_33799, PMID: 37782189

[75] Butler AE Ramanjaneya M Moin ASM Hunt SC Atkin SL . Clinical improvement may not reflect metabolic homeostasis normalization in subjects with and without roux-en-y bariatric surgery after 12 years: comparison of surgical subjects to a lean cohort. Front Endocrinol. (2023) 14:1228853. doi: 10.3389/fendo.2023.1228853, PMID: 37810875 PMC10552523

[76] Ji S Park SJ Lee JY Baek JY Jung HW Kim K . Lack of association between serum myonectin levels and sarcopenia in older asian adults. Exp Gerontol. (2023) 178:112229. doi: 10.1016/j.exger.2023.112229, PMID: 37270069

[77] Xue S Ling J Tian M Li K Li S Liu D . Combined serum CTRP7 and CTRP15 levels as a novel biomarker for insulin resistance and type 2 diabetes mellitus. Heliyon. (2024) 10:e30029. doi: 10.1016/j.heliyon.2024.e30029, PMID: 38726186 PMC11078869

[78] Ismail ZM Al-Fartusie FS Tahir NT Ismail AH . Investigation the relationship between myonectin levels and both lipid profiles and liver function tests in diabetic nephropathy patients. J Port Sci Res. (2024) 7:30–5. doi: 10.36371/port.2024.1.5

[79] Tuama R Sarhat E . The role of myonectin in patients with type 2 diabetes mellitus. Georgian Med News. (2024) 351):96–9., PMID: 39230229

[80] Sheptulina AF Mamutova EM Elkina AY Timofeev YS Metelskaya VA Kiselev AR . Serum irisin, myostatin, and myonectin correlate with metabolic health markers, liver disease progression, and blood pressure in patients with metabolic dysfunction-associated fatty liver disease and hypertension. Metabolites. (2024) 14:584. doi: 10.3390/metabo14110584, PMID: 39590820 PMC11596689

[81] Joy JM Vogel RM Moon JR Falcone PH Mosman MM Pietrzkowski Z . Ancient peat and apple extracts supplementation may improve strength and power adaptations in resistance trained men. BMC Complement Altern Med. (2016) 16:224. doi: 10.1186/s12906-016-1222-x, PMID: 27430755 PMC4950767

[82] Sabouri M Taghibeikzadehbadr P Shabkhiz F Izanloo Z Shaghaghi FA . Effect of eccentric and concentric contraction mode on myogenic regulatory factors expression in human vastus lateralis muscle. J Muscle Res Cell Motil. (2022) 43:9–20. doi: 10.1007/s10974-021-09613-x, PMID: 35018575

[83] Rezaeimanesh D . The effect of a period of high-intensity interval swimming training on plasma levels of myonectin, insulin-like growth factor-1 (IGF-1), and lipid profile in overweight men. Int J Chem Biochem Sci. (2022) 22:86–92.

[84] Petro JL Fragozo-Ramos MC Milán AF Aristizabal JC Calderón JC Gallo-Villegas J . Efficacy of high-intensity interval training versus continuous training on serum myonectin and lipid outcomes in adults with metabolic syndrome: A *post-hoc* analysis of a clinical trial. PloS One. (2024) 19:e0307256. doi: 10.1371/journal.pone.0307256, PMID: 39024345 PMC11257237

[85] Cairns SP Borrani F . B-adrenergic modulation of skeletal muscle contraction: key role of excitation-contraction coupling. J Physiol. (2015) 593:4713–27. doi: 10.1113/jp270909, PMID: 26400207 PMC4626548

[86] Bolaños P Calderón JC . Excitation-contraction coupling in mammalian skeletal muscle: blending old and last-decade research. Front Physiol. (2022) 13:989796. doi: 10.3389/fphys.2022.989796, PMID: 36117698 PMC9478590

[87] Subbotina E Sierra A Zhu Z Gao Z Koganti SR Reyes S . Musclin is an activity-stimulated myokine that enhances physical endurance. Proc Natl Acad Sci U.S.A. (2015) 112:16042–7. doi: 10.1073/pnas.1514250112, PMID: 26668395 PMC4702977

[88] Calle-Ciborro B Espin-Jaime T Santos FJ Gomez-Martin A Jardin I Pozo MJ . Secretion of interleukin 6 in human skeletal muscle cultures depends on CA^2+^ signalling. Biol (Basel). (2023) 12(7):968. doi: 10.3390/biology12070968, PMID: 37508398 PMC10376320

[89] Besse-Patin A Montastier E Vinel C Castan-Laurell I Louche K Dray C . Effect of endurance training on skeletal muscle myokine expression in obese men: identification of apelin as a novel myokine. Int J Obes (Lond). (2014) 38:707–13. doi: 10.1038/ijo.2013.158, PMID: 23979219

[90] Wright DC Geiger PC Han DH Jones TE Holloszy JO . Calcium induces increases in peroxisome proliferator-activated receptor gamma coactivator-1alpha and mitochondrial biogenesis by a pathway leading to P38 mitogen-activated protein kinase activation. J Biol Chem. (2007) 282:18793–9. doi: 10.1074/jbc.M611252200, PMID: 17488713

[91] Freyssenet D Di Carlo M Hood DA . Calcium-dependent regulation of cytochrome C gene expression in skeletal muscle cells. Identification of a protein kinase C-dependent pathway. J Biol Chem. (1999) 274:9305–11. doi: 10.1074/jbc.274.14.9305, PMID: 10092607

[92] Ojuka EO Jones TE Han DH Chen M Wamhoff BR Sturek M . Intermittent increases in cytosolic CA^2+^ Stimulate mitochondrial biogenesis in muscle cells. Am J Physiol Endocrinol Metab. (2002) 283:E1040–5. doi: 10.1152/ajpendo.00242.2002, PMID: 12376333

[93] Lee H Cashin AG Lamb SE Hopewell S Vansteelandt S VanderWeele TJ . A guideline for reporting mediation analyses of randomized trials and observational studies: the agrema statement. Jama. (2021) 326:1045–56. doi: 10.1001/jama.2021.14075, PMID: 34546296 PMC8974292

[94] Hou N Du G Han F Zhang J Jiao X Sun X . Irisin regulates heme oxygenase-1/adiponectin axis in perivascular adipose tissue and improves endothelial dysfunction in diet-induced obese mice. Cell Physiol Biochem. (2017) 42:603–14. doi: 10.1159/000477864, PMID: 28595178

[95] Kubrak O Jorgensen AF Koyama T Lassen M Nagy S Hald J . LGR signaling mediates muscle-adipose tissue crosstalk and protects against diet-induced insulin resistance. Nat Commun. (2024) 15:6126. doi: 10.1038/s41467-024-50468-w, PMID: 39033139 PMC11271308

[96] Sirek T Sirek A Oplawski M Boron D Chalcarz M Ossowski P . Expression profile of messenger and micro RNAs related to the histaminergic system in patients with five subtypes of breast cancer. Front Oncol. (2024) 14:1407538. doi: 10.3389/fonc.2024.1407538, PMID: 39267843 PMC11390352

[97] Coffey R Jung G Olivera JD Karin G Pereira RC Nemeth E . Erythroid overproduction of erythroferrone causes iron overload and developmental abnormalities in mice. Blood. (2022) 139:439–51. doi: 10.1182/blood.2021014054, PMID: 34614145 PMC8777203

[98] Kautz L Jung G Valore EV Rivella S Nemeth E Ganz T . Identification of erythroferrone as an erythroid regulator of iron metabolism. Nat Genet. (2014) 46:678–84. doi: 10.1038/ng.2996, PMID: 24880340 PMC4104984

[99] Barbalho SM Laurindo LF Tofano RJ Flato UAP Mendes CG de Alvares Goulart R . Dysmetabolic iron overload syndrome: going beyond the traditional risk factors associated with metabolic syndrome. Endocrines. (2023) 4:18–37. doi: 10.3390/endocrines4010002

[100] Vaughan RA Gannon NP Barberena MA Garcia-Smith R Bisoffi M Mermier CM . Characterization of the metabolic effects of irisin on skeletal muscle *in vitro* . Diabetes Obes Metab. (2014) 16:711–8. doi: 10.1111/dom.12268, PMID: 24476050

[101] Seldin MM Tan SY Wong GW . Metabolic function of the CTRP family of hormones. Rev Endocr Metab Disord. (2014) 15:111–23. doi: 10.1007/s11154-013-9255-7, PMID: 23963681 PMC3931758

[102] Balasubramanian P Schaar AE Gustafson GE Smith AB Howell PR Greenman A . Adiponectin receptor agonist adiporon improves skeletal muscle function in aged mice. Elife. (2022) 11:e71282. doi: 10.7554/eLife.71282, PMID: 35297761 PMC8963882

[103] Yamauchi T Kamon J Minokoshi Y Ito Y Waki H Uchida S . Adiponectin stimulates glucose utilization and fatty-acid oxidation by activating amp-activated protein kinase. Nat Med. (2002) 8:1288–95. doi: 10.1038/nm788, PMID: 12368907

[104] Hawley JA Lessard SJ . Exercise training-induced improvements in insulin action. Acta Physiol (Oxf). (2008) 192:127–35. doi: 10.1111/j.1748-1716.2007.01783.x, PMID: 18171435

[105] Sierra APR Fontes-Junior AA Paz IA de Sousa CAZ Manoel L Menezes DC . Chronic low or high nutrient intake and myokine levels. Nutrients. (2022) 15:153. doi: 10.3390/nu15010153, PMID: 36615810 PMC9824657

[106] Gallo-Villegas J Castro-Valencia LA Pérez L Restrepo D Guerrero O Cardona S . Efficacy of high-intensity interval- or continuous aerobic-training on insulin resistance and muscle function in adults with metabolic syndrome: A clinical trial. Eur J Appl Physiol. (2022) 122:331–44. doi: 10.1007/s00421-021-04835-w, PMID: 34687360

