# Supplementary material for: Myonectin and metabolic health: a systematic review
Source: Front Endocrinol (Lausanne). 2025 Jul 16;16:1557142. doi: 10.3389/fendo.2025.1557142 (PMC12307190; doi:10.3389/fendo.2025.1557142)
Supplement: Supplementary file 1 [file DataSheet1.docx]

Myonectin and Metabolic Health: A Systematic Review

Jorge L. Petro^1,2*^, Jaime Gallo-Villegas^3,4^, Juan C. Calderón^1*^

^1^ Physiology and Biochemistry Research Group-PHYSIS, Faculty of Medicine, University of Antioquia, Medellín, Colombia.

^2^ Research Group in Physical Activity, Sports and Health Sciences (GICAFS), Universidad de Córdoba, Montería, Colombia.

^3^ Research Group in Medicine Applied to Physical Activity and Sports-GRINMADE, Medellín, Colombia.

^4^ Centro Clínico y de Investigación SICOR, Medellín, Colombia.

**^*^ Correspondence:**

[jorgelpetro@correo.unicordoba.edu.co](mailto:jorgelpetro@correo.unicordoba.edu.co) (J.L. Petro.); [jcalderonv00@yahoo.com](mailto:jcalderonv00@yahoo.com) (J.C. Calderón).

Supplementary Material


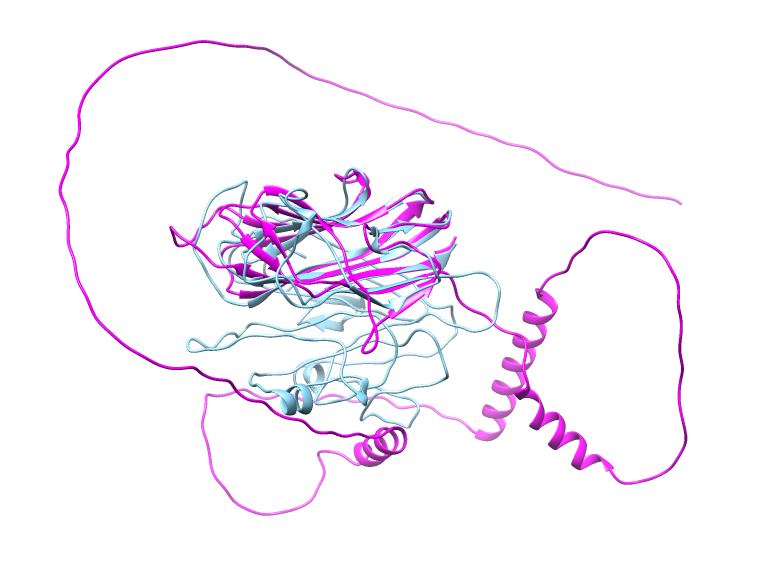


**Supplementary Figure 1.** The human myonectin structure predicted by AlphaFold (magenta) and I-TASSER (blue) was compared, yielding a root mean square deviation (RMSD) of 0.821 Å across 70 pruned atom pairs. However, when considering all 354 atom pairs, the RMSD increased significantly to 37.758 Å. The overlay highlights both the similarities and differences between the models. The structural comparison was conducted using ChimeraX, employing the Needleman-Wunsch alignment algorithm with the BLOSUM-62 similarity matrix and specific gap penalties for alignment.

| **miRNA** | **Score** |
| --- | --- |
| hsa-miR-4251 | 93 |
| hsa-miR-450a-2-3p | 91 |
| hsa-miR-3619-5p | 91 |
| hsa-miR-214-3p | 85 |
| hsa-miR-7854-3p | 85 |
| hsa-miR-210-5p | 85 |
| hsa-miR-761 | 85 |
| hsa-miR-148a-5p | 85 |
| hsa-miR-2467-3p | 83 |
| hsa-miR-486-5p | 82 |

**Supplementary Table 1. Human miRNA predicted to target human *ERFE* in miRDB.** ^a^miRNA, micro ribonucleic acid; *ERFE*, erythroferrone gene; miRDB, miRNA database. Accessed on May 29^th^, 2025.


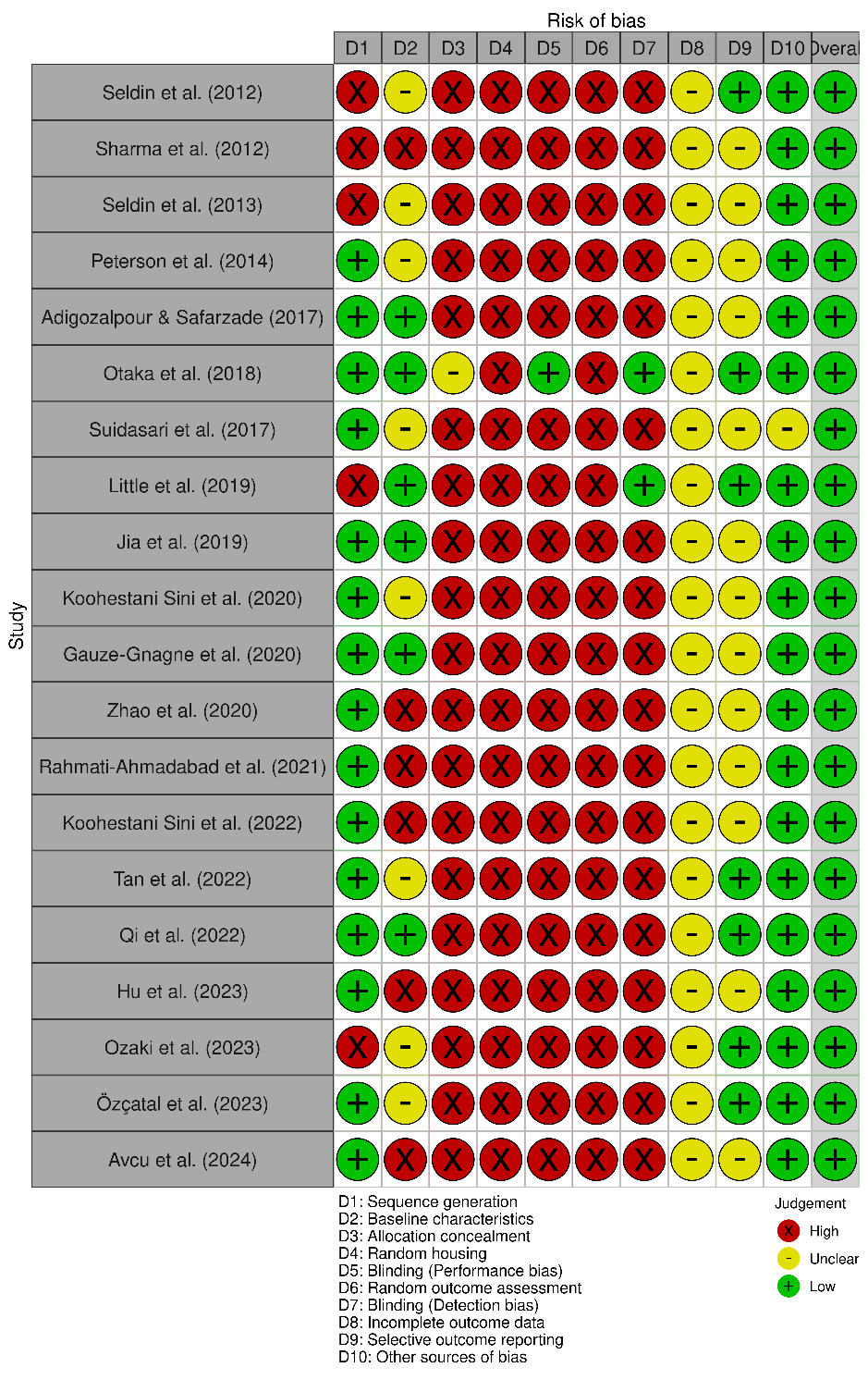


**Supplementary Figure 2. Risk of bias in individual animal studies assessed using the SYRCLE tool.**


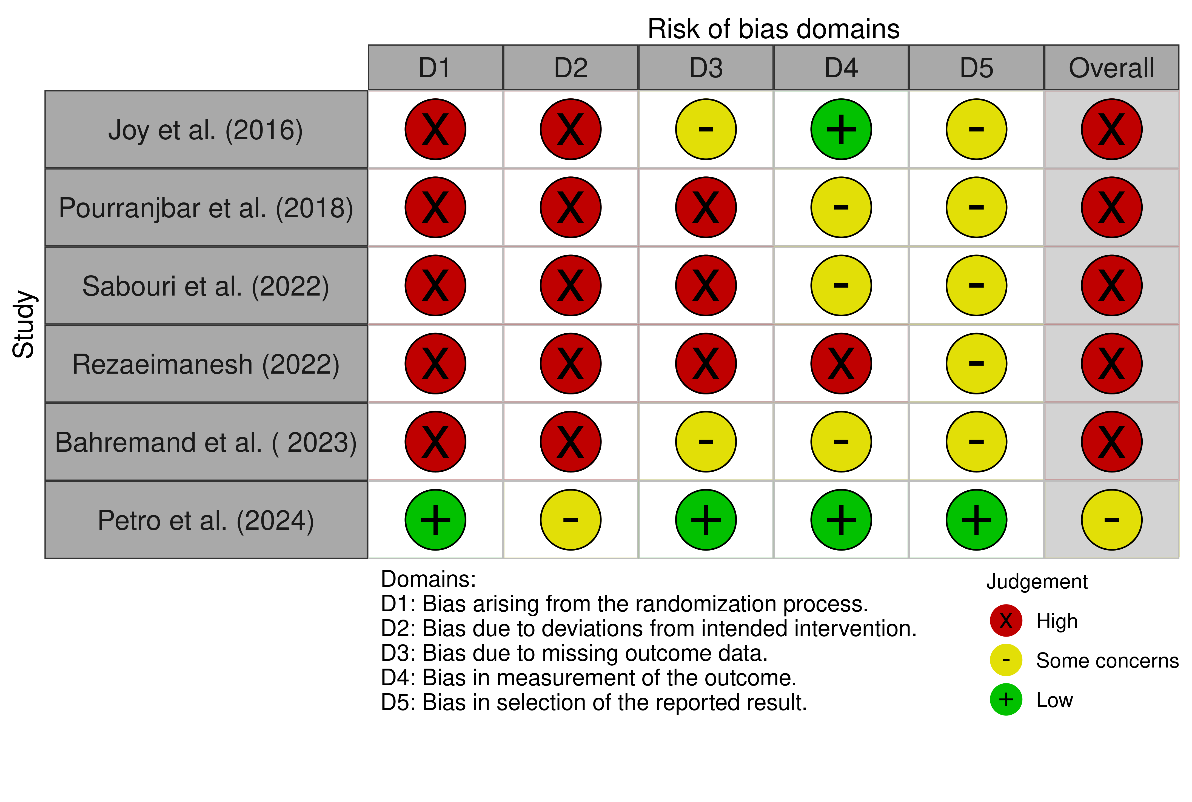


**Supplementary Figure 3. Risk of bias in individual randomized controlled trials assessed using the RoB 2 tool.**
